# Community Health Worker Support for Hispanic and Latino Individuals Receiving Hemodialysis: The Navigate-Kidney Randomized Clinical Trial

**DOI:** 10.1001/jamainternmed.2025.5305

**Published:** 2025-11-07

**Authors:** Lilia Cervantes, Elizabeth Juarez-Colunga, Neil R. Powe, Jennifer E. Flythe, John F. Steiner, Daniel Cukor, Romana Hasnain-Wynia, Seth Furgeson, Ladan Golestaneh, Claudia Camacho, Lauren McBeth, Brenda L. Beaty, Jiayuan Shi, Emily Bacon, Michel Chonchol

**Affiliations:** 1 Department of Medicine, University of Colorado Anschutz Medical Center, Aurora; 2 Department of Biostatistics and Informatics, University of Colorado Anschutz Medical Campus, Aurora; 3 Priscilla Chan and Mark Zuckerberg San Francisco General Hospital, University of California, San Francisco; 4 Division of Nephrology and Hypertension, University of North Carolina School of Medicine, Chapel Hill; 5 Cecil G. Sheps Center for Health Services Research, University of North Carolina, Chapel Hill; 6 Departments of Medicine and Psychiatry, New York University Grossman School of Medicine, New York, New York; 7 Division of Renal Diseases and Hypertension, University of Colorado Anschutz Medical Campus, Aurora; 8 Department of Medicine, Denver Health Hospital, Denver, Colorado; 9 Section of Nephrology, Department of Medicine, Yale University School of Medicine, New Haven, Connecticut; 10 Adult and Child Center for Outcomes Research and Delivery Science (ACCORDS), University of Colorado Anschutz Medical Campus, Aurora; 11 Bacon Analytics, Denver, Colorado

## Abstract

**Importance:**

Hispanic and Latino individuals with hemodialysis-dependent kidney failure experience social barriers that may increase their risk for volume-related complications, including fluid accumulation between dialysis sessions or interdialytic weight gain (IDWG). Higher IDWG contributes to cardiovascular complications, hospitalizations, and poor quality of life. Minimizing IDWG is prioritized by patients, clinicians, and payers; yet, high IDWG remains common despite dietary education.

**Objective:**

To determine if community health worker (CHW) support reduces IDWG among Hispanic and Latino individuals receiving hemodialysis.

**Design, Setting, and Participants:**

This parallel group, unblinded, multicenter, community-partnered, randomized clinical trial was conducted at 5 dialysis centers in Denver, Colorado, between November 2020 and August 2022. Self-identified Latino or Hispanic adults receiving in-center hemodialysis were eligible. Data were analyzed from August 2024 to July 2025.

**Intervention:**

Participants were randomized 1:1 to the Navigate-Kidney intervention or standard care. In Navigate-Kidney, CHWs followed a framework-driven, patient-centered protocol to support patient social needs and provide health navigation and culturally responsive kidney care education, including dietary modification and fluid restriction education. CHWs met participants biweekly for at least 6 visits. Standard care had no trial-driven culturally responsive components.

**Main Outcomes and Measures:**

The primary outcome was change in IDWG from 90 days prior to intervention to 180 days postintervention, calculated as a percentage of estimated dry weight. Secondary outcomes included missed and shortened dialysis sessions, health care utilization, and patient activation. Between-group IDWG differences were estimated with piecewise linear mixed models.

**Results:**

Of 139 participants (mean [SD] age, 56.8 [12.9] years; 68 [49%] female), 68 were randomly assigned to Navigate-Kidney and 71 to standard care. Postintervention trends in percentage of estimated dry weight differed between Navigate-Kidney and standard care, which corresponded to a difference between groups of −0.46 percentage points (95% CI, −0.78 to −0.14 percentage points) in IDWG after 180 days of follow-up (*P* = .01). Mean IDWG was 3.26% (95% CI, 2.83%-3.68%) and 3.72% (95% CI, 3.30%-4.14%) in Navigate-Kidney and standard care, respectively. There were fewer shortened dialysis sessions with Navigate-Kidney vs SC (median [IQR], 0.1 [−1.2 to 1.1] vs 0.6 [−0.5 to 1.8]; *P* = .02), as well as greater improvement in the Patient Activation Measure (median [IQR], 1.8 [−2.2 to 5.2] points vs −2.2 [−7.4 to 2.5] points; *P* = .005).

**Conclusions and Relevance:**

In this randomized clinical trial, a culturally tailored CHW intervention modestly lowered IDWG and improved dialysis adherence and patient activation among Hispanic and Latino patients with hemodialysis-dependent kidney failure.

**Trial Registration:**

ClinicalTrials.gov Identifier: NCT03978806

## Key Points

**Question:**

Does providing a community health worker (CHW)–based intervention for dietary education and self-efficacy improve interdialytic weight gain (IDWG) among Hispanic and Latino individuals with hemodialysis-dependent kidney failure?

**Findings:**

In this randomized clinical trial that included 139 patients at 5 dialysis centers in Denver, Colorado, from 2020 to 2022, a CHW intervention modestly reduced IDWG compared to standard care. There were fewer shortened dialysis sessions with the intervention and greater improvement in patient activation.

**Meaning:**

Providing CHW support for Hispanic and Latino individuals receiving hemodialysis is a practical and meaningful approach to improving patient outcomes, including lowering IDWG.

## Teaser

This randomized clinical trial evaluates if community health worker support reduces interdialytic weight gain among Hispanic and Latino individuals receiving hemodialysis.

## Introduction

Hispanic and Latino individuals with hemodialysis-dependent kidney failure face a disproportionate burden of multilevel social and structural challenges that contribute to kidney health disparities—including at the individual (low patient activation, poverty, poor access to healthy foods), interpersonal (lack of culturally responsive care), community (distrust, food deserts), and societal (immigration policies, exclusion from health insurance) levels.^1,2,3,4,5,6,7,8,9,10,11,12,13,14,15,16,17,18,19,20,21,22,23^ Despite these challenges and when asked what would improve their well-being, Hispanic and Latino individuals consistently desired to improve their knowledge about kidney disease and dialysis care, especially dietary restrictions.^2,13,21,24^ This knowledge is critically important because dietary restriction adherence is the most effective strategy for lowering interdialytic weight gain (IDWG; ie, accumulated fluid between dialysis sessions). Large IDWGs contribute to chronic volume overload and are associated strongly with higher risks of cardiovascular hospitalizations and mortality.^25,26^

Individuals receiving hemodialysis have a 10- to 20-fold higher risk of cardiovascular death than individuals not receiving hemodialysis, and cardiovascular death is the leading cause of death in the hemodialysis population. Systematic reviews of clinical trials demonstrate that dietary self-management lowers IDWG.^27,28^ Reducing IDWG is a particular challenge, as it necessitates knowledge of dietary restrictions.^29,30,31^ Evidence suggests that involvement of community health workers (CHWs; ie, navigators) can improve diet and other person-centered outcomes for other chronic conditions.^32,33,34,35,36,37,38,39,40,41,42^ CHWs are culturally and language concordant and provide culturally tailored education, promote self-efficacy, facilitate interactions between patients and the health care system, and can help overcome modifiable social barriers, such as access to healthy foods.^8,17^ To our knowledge, no prior study has evaluated a CHW-based intervention for dietary education and self-efficacy among Hispanic and Latino individuals with hemodialysis-dependent kidney failure.

To address the preferences and needs of Hispanic and Latino individuals with hemodialysis-dependent kidney failure, this community-partnered research team codeveloped and tested Navigate-Kidney, a CHW-delivered intervention for Hispanic and Latino individuals receiving in-center hemodialysis. In a single-arm study, we previously demonstrated the feasibility and acceptability of Navigate-Kidney.^6,8,17^ In the current randomized clinical trial, we evaluate the effect of Navigate-Kidney vs standard care dietary education on IDWG among Hispanic and Latino individuals receiving hemodialysis.

## Methods

### Trial Design

We conducted a parallel-group randomized clinical trial to determine whether Navigate-Kidney reduces IDWG compared to standard care (Supplement 1). The University of Colorado institutional review board approved the study. Participants provided written informed consent and received remuneration at enrollment and study end. We followed the Consolidated Standards of Reporting Trials (CONSORT) reporting guidelines in reporting this study.^43^

### Participants and Settings

Participants were enrolled by an experienced CHW at 5 urban dialysis centers in Denver, Colorado, between October 2020 and April 2022, then followed up through August 2022. English- or Spanish-speaking adults (≥18 years old) who self-identified as Latino or Hispanic and received thrice-weekly hemodialysis for 3 months or longer were eligible to participate. Individuals were excluded if they had cognitive impairment, active suicidal intent, psychosis, bipolar disorder, pregnancy, or were expected to undergo kidney transplantation within 3 months. Information about historical IDWG patterns did not affect study eligibility. The research team partnered with a community steering committee involved in the initial development and testing of Navigate-Kidney and met bimonthly.

### Randomization

We randomized participants stratified by site to the intervention or standard care using a 1:1 ratio. Blocked randomization based on random permuted blocks of sizes 2, 4, and 6 was used. Blinding was not possible due to the nature of the intervention.

### Interventions

Participants assigned to Navigate-Kidney were seen by a CHW within 2 weeks of enrollment and then every 2 weeks for a total of 6 or more visits within the first 3 to 4 months of enrollment. The duration of the intervention varied over the course of the study because additional CHW visits (beyond the 6 required visits) were allowed during the COVID-19 pandemic due to its additional social challenges. The intervention concluded when no additional CHW visits were requested within 2 weeks of the last CHW visit. Visits took place at the dialysis center or at a location selected by the participant. As shown in eFigure 1 in Supplement 2, CHWs were trained and supervised to emphasize 4 core intervention functions (termed functions by the form-function framework)^44^: (1) build trust through understanding of health experience, (2) address multilevel social and structural challenges to facilitate health system navigation, (3) provide patient-centered education, and (4) enhance self-management. To support health navigation, the CHW acted as a cultural broker, meeting with the dialysis center dietitian alongside the participant at least once. Participants assigned to standard care had no trial-driven culturally responsive components and received standard dietary education from a dialysis center dietitian.

### Effect of the COVID-19 Pandemic

The study was scheduled to launch in March 2020 with a primary outcome of depressive affect. However, due to the onset of the COVID-19 pandemic and state policy changes affecting the dialysis care of Hispanic and Latino individuals, there was concern that many patients might have depressive affect recalcitrant to the study intervention. As such, when the study launched in October 2020, the primary outcome was modified to IDWG. In addition, to account for the new social challenges faced by participants during the pandemic, the number of study CHW visits was increased from a maximum of 6 to unlimited if requested by participants.

### Outcomes and Follow-Up

The primary outcome was change in IDWG, measured on a session-to-session basis, in a 90-day period prior to randomization and then during a 180-day period following intervention end. IDWG was calculated as the difference in standing weight at the beginning of each hemodialysis session (preweight) minus the standing weight after (postweight) the previous hemodialysis session, divided by the prescribed estimated dry weight, expressed as the percentage of change (IDWG = [preweight − postweight]/estimated dry weight,%).

Secondary outcomes included hemodialysis adherence (eg, shortened treatments and missed treatments), health care utilization, patient-reported outcomes, and socioeconomic challenges. Secondary outcome definitions and instrument descriptions are provided in eTable 1 in Supplement 2.^45,46,47,48,49,50^

### Data Collection

Patient characteristics, including demographic, comorbid medical, and socioeconomic information; dialysis treatment–related variables; laboratory values; and health care utilization, were extracted from the large dialysis organization electronic health record. Health literacy (assessed with the 4-item Brief Health Literacy Screening tool)^51,52^ and patient-reported outcomes were obtained directly from participants.

### Sample-Size Calculations

Based on previous clinical trials, the estimated effect sizes of IDWG ranged from 0.5 to 0.7.^45,53,54,55^ We assumed a conservative target effect size of 0.50. We determined that a sample size of 128 participants would provide 80% power to detect a difference of 0.5 SD units using a 2-sample *t* test to compare changes from baseline to the end of the study in IDWG between treatment and standard care. There were some inconsistencies in the protocol regarding adjustment for dropout rates of 10% and 20%. However, the study ultimately used a target sample size of 141 participants, which adjusted for a 9% dropout rate (eg, due to clinic transfer, modality change, death).

### Statistical Analysis

To analyze the primary outcome, the protocol originally specified using an analysis of covariance model, with IDWG at the end of the intervention as the outcome, adjusting for baseline IDWG. However, before analyzing the data, we updated the analysis plan to use a linear mixed model, which incorporates all available IDWG data, accounts for data missing at random, and offers a more powerful approach. To evaluate changes in IDWG over time, we modeled the primary outcome using a linear mixed model with a knot at the start of the intervention, which we refer to as a piecewise linear mixed model (PLMM). A knot in a linear regression model is a time point at which the behavior (trend) of the model can change. We adjusted for enrollment site as a fixed effect and included random intercepts for participants and random slopes for days (using lmer from lme4 package, version 1.1-34, and lmerTest package, version 3.1-3, in R [R Project for Statistical Computing]). This model accounted for different mean trajectories after the start of the intervention period. Because some participants had longer intervention periods than others (ie, due to requesting additional CHW visits during the pandemic) and to align trajectories after the intervention period, we removed data collected during delivery of the intervention from the primary analysis. Since the study was randomized, we assumed that there was no difference in the trends (slopes) of IDWG in the 90-day preintervention period. This assumption was then tested in a sensitivity analysis. The primary hypothesis was tested using a likelihood ratio test, comparing the models with and without an interaction between time and intervention group, which allows for different trends/slopes in the postintervention period. We used appropriate contrasts to estimate differences in the preintervention and postintervention periods at different time points (with emmeans from emmeans package, version 1.8.8, in R [R Project for Statistical Computing]). The assumption of no difference in the slopes in the preintervention period was examined through both a sensitivity analysis using a PLMM that allowed for different slopes in the preintervention period but still tested the effect of the intervention in the postintervention period, and using local polynomial regression to nonparametrically estimate the mean of IDWG over time (performed using loess R function from stats package, version 4.3.1 [R Project for Statistical Computing]). Assumptions, including normality, constant variance, and linearity, of the PLMM were examined. See eTable 2 in Supplement 2 for additional statistical analyses.

We analyzed secondary outcomes as follows: rates of missed and shortened dialysis sessions were analyzed (separately) using the Wilcoxon rank-sum test, predialysis sitting systolic blood pressure and phosphorus longitudinal values were analyzed using a PLMM adjusting for enrollment site, health care utilization was analyzed using exact conditional logistic regression, and patient-reported outcomes were analyzed using the Wilcoxon rank-sum test. Social risks, recorded at baseline and at the last study visit, were compared between study groups using logistic regression. For each social risk, the binary outcome was whether the situation had improved or not (0/1) from baseline to the last study visit.

A subgroup analysis was conducted with the subset of participants with low baseline patient activation. We used SAS, version 9.4 (SAS Institute) for raw data cleaning and analyses of health care utilization, and R software, version 4.4.2 (R Project for Statistical Computing), for all the other analyses. A 2-sided *P* value of .05 was considered statistically significant. Data were analyzed from August 2024 to July 2025.

## Results

### Participant Characteristics

Of 174 participants assessed for eligibility (Figure), 12 did not meet study inclusion criteria, and 23 declined to participate. Reasons for declining included doing well and having enough support (n = 11), other health issues (n = 4), and no reason given (n = 8). Of the 139 participants enrolled, 68 were assigned to Navigate-Kidney and 71 to standard care. No participants were lost to follow-up; at least partial data were available for all participants. The median (IQR) follow-up period after the intervention was 182 (179-183) days and was similar in the 2 treatment groups. Even though the intervention was designed to deliver at least 6 visits to each participant, participants had a range of visits from 4 through 19, with a median (IQR) of 6.5 (5-8) visits.

**Figure.**
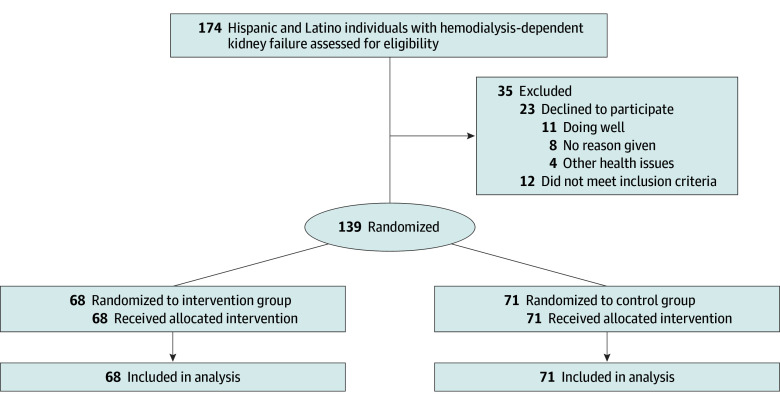
Consort Flow Diagram

The 139 included participants (mean [SD] age, 56.8 [12.9] years; 68 [49%] female) had a median (IQR) dialysis duration of 34 (12-64) months, 95 (68%) reported limited English proficiency, 103 (74%) had less than high school education, and 96 (72%) had household income below $25 000. Thirty-nine participants (28%) experienced housing insecurity, and 35 (25%) and 42 (30%) reported food or health care access issues in the past year, respectively. Food insecurity in the form of food running out was reported as often or sometimes true by 58 participants (42%) and as inability to afford balanced meals by 68 participants (49%) (Table 1).

**Table 1.** Characteristics of the Study Population

Characteristic	No. (%)		
	Overall (N = 139)	Intervention (n = 68)	Standard care (n = 71)
Age, mean (SD), y	57 (13)	58 (12)	56 (13)
Sex			
Female	68 (49)	38 (56)	30 (42)
Male	71 (51)	30 (44)	41 (58)
Self-identified Hispanic or Latino	139 (100)	68 (100)	71 (100)
Self-identified and self-reported race			
American Indian or Alaska Native	8 (6)	6 (9)	2 (3)
Native Hawaiian	1 (1)	0	1 (1)
White	21 (15)	11 (16)	10 (14)
Latino/Hispanic or no race	109 (78)	51 (75)	58 (82)
Country of origin			
Mexico	106 (76)	49 (72)	57 (80)
Other (El Salvador, Honduras, Peru)	6 (4)	4 (6)	2 (3)
US	27 (19)	15 (22)	12 (17)
In general, read and speak language in Spanish	114 (82)	55 (81)	59 (83)
How well do you speak English?			
Very well	26 (19)	13 (19)	13 (18)
Well	18 (13)	10 (15)	8 (11)
Not well	61 (44)	28 (41)	33 (46)
Not at all	34 (24)	17 (25)	17 (24)
Highest level of school finished			
More than high school	20 (14)	11 (16)	9 (13)
High school diploma or General Educational Development certificate	16 (12)	7 (10)	9 (13)
Less than high school	103 (74)	50 (74)	53 (75)
Current work situation^a^			
Full-time work	5 (4)	2 (3)	3 (4)
Part-time or temporary work	20 (15)	4 (6)	16 (23)
Unemployed	111 (82)	60 (91)	51 (73)
Past year total combined income for household, $^b^			
<25 000	96 (72)	48 (73)	48 (71)
≥25 000	22 (16)	12 (18)	10 (15)
Do not know/choose not to answer	16 (12)	6 (9)	10 (15)
Insurance			
Dual Medicare/Medicaid	47 (34)	24 (35)	23 (32)
Medicaid	34 (24)	20 (29)	14 (20)
Medicare	6 (4)	3 (4)	3 (4)
Other public or private	52 (37)	21 (31)	31 (44)
In past year, household members unable to get:			
Food	35 (25)	18 (26)	17 (24)
Clothing	32 (23)	16 (24)	16 (23)
Utilities	48 (35)	27 (40)	21 (30)
Childcare	7 (5)	5 (7)	2 (3)
Medicine or any health care	42 (30)	22 (32)	20 (28)
Cell phone	29 (21)	16 (24)	13 (18)
In past 12 mo, electric, gas, oil, or water company threatened to shut off services^c^	25 (18)	11 (16)	14 (20)
In past 12 mo, there were days that home was not heated/cooled because bills were not paid	11 (8)	7 (10)	4 (6)
In past 12 mo, how many times was it decided not to fill or refill a prescription because it was too expensive			
1	15 (11)	7 (10)	8 (11)
2	13 (9)	7 (10)	6 (8)
3-4	17 (12)	7 (10)	10 (14)
None	94 (68)	47 (69)	47 (66)
Mode of transport to and from dialysis^c^			
Benefit transportation (Medicaid)	38 (28)	17 (25)	21 (30)
Family or friend	35 (26)	21 (31)	14 (20)
Drive self	48 (35)	18 (27)	30 (43)
Public transportation	16 (12)	11 (16)	5 (7)
Within past 12 mo, we worried whether our food would run out before we got money to buy more			
Never true	81 (58)	41 (60)	40 (56)
Often true	11 (8)	6 (9)	5 (7)
Sometimes true	47 (34)	21 (31)	26 (37)
Within past 12 mo, food bought did not last and there was not money to get more			
Never true	87 (63)	44 (65)	43 (61)
Often true	12 (9)	7 (10)	5 (7)
Sometimes true	40 (29)	17 (25)	23 (32)
Within past 12 mo, could not afford to eat balanced meals			
Never true	71 (51)	35 (51)	36 (51)
Often true	12 (9)	4 (6)	8 (11)
Sometimes true	56 (40)	29 (43)	27 (38)
Worried about losing housing	39 (28)	19 (28)	20 (28)
Patient Activation Measure level			
1: Disengaged and overwhelmed	25 (18)	14 (21)	11 (15)
2: Becoming aware but struggling	49 (35)	24 (35)	25 (35)
3: Taking action	33 (24)	15 (22)	18 (25)
4: Maintaining behaviors and pushing further	32 (23)	15 (22)	17 (24)
Months receiving dialysis, median (IQR)	34.0 (12.0-64.0)	34.5 (12.0-63.0)	30.0 (12.0-66.0)
Distance between home and dialysis center, median (IQR), miles^c^	5.0 (3.0-8.0)	5.0 (3.0-8.0)	5.0 (3.0-7.8)
Travel time between home and dialysis center, median (IQR), min^c^	15.0 (10.0-25.0)	15.0 (10.0-25.0)	15.0 (10.0-20.0)
Kidney Disease Quality of Life instrument, mean (SD)			
Kidney summary score^d^	65.4 (17.0)	62.9 (19.0)	67.7 (14.5)
Burden of kidney disease	35.9 (23.1)	34.1 (24.5)	37.6 (21.6)
Symptoms/problems	74.0 (16.8)	71.4 (18.4)	76.5 (14.8)
Effects of kidney disease	67.1 (23.4)	64.4 (25.1)	69.7 (21.4)
Short form 12: physical health composite	35.0 (9.8)	32.5 (8.9)	37.4 (10.0)
Short form 12: mental health composite	50.6 (10.2)	50.3 (10.5)	50.9 (10.0)
Brief Health Literacy score, mean (SD)	9.7 (5.0)	9.3 (5.1)	10.0 (4.9)
No. of family members currently living in same household, median (IQR)	3.0 (2.0-5.0)	3.0 (2.0-5.0)	3.0 (2.0-4.5)
Laboratory measurements			
Serum albumin, mean (SD), g/dL^e^	4 (0.5)	4 (0.6)	4.1 (0.4)
Serum potassium, mean (SD), mEq/L^f^	4.8 (0.8)	4.9 (0.9)	4.7 (0.7)
Serum phosphorus, mean (SD), mg/dL^b^	5.5 (1.9)	5.5 (2.1)	5.6 (1.7)
Charlson Comorbidity Index, mean (SD)^g^	2.8 (1.7)	3.0 (1.9)	2.7 (1.6)
Follow-up period after the intervention, median (IQR), d	182.0 (179.0-182.5)	182.0 (176.5-183.0)	181.0 (179.0-182.0)
Enrollment site			
1	18 (13)	5 (7)	13 (18)
2	31 (22)	18 (26)	13 (18)
3	50 (36)	23 (34)	27 (38)
4	26 (19)	14 (21)	12 (17)
5	14 (10)	8 (12)	6 (8)

SI conversion factor: to covert serum albumin to g/L, multiply by 10; serum potassium to mmol/L, multiply by 1; and serum phosphorus to mmol/L, multiply by 0.323.

^a^ Data missing for 3 participants.

^b^ Data missing for 5 participants.

^c^ Data missing for 2 participants.

^d^ Higher scores mean better quality of life.

^e^ Data missing for 24 participants.

^f^ Data missing for 6 participants.

^g^ Data missing for 51 participants.

Baseline patient activation was low (level 1 or 2) in 74 participants (53%). Health-related quality of life was measured with the Kidney Disease Quality of Life instrument, and mean (SD) scores were 65.4 (17.0) for the overall score, 35.0 (9.8) for the physical health composite, and 50.6 (10.2) for the mental health composite.

### Primary Outcome

There was a statistically significant difference in postintervention trends (slopes) of IDWG between the intervention and standard care (*P* = .01; Table 2). This trend corresponded to a difference of −0.46 percentage points [pp] (95% CI, −0.78 to −0.14 pp) in IDWG at the end of the follow-up between Navigate-Kidney vs standard care (mean IDWG, 3.26% [95% CI, 2.83%-3.68%] vs 3.72% [95% CI, 3.30%-4.14%]). The sensitivity analysis allowing IDWG trends to differ before the start of intervention showed a statistically significant intervention effect (*P* = .004; eTable 3 in Supplement 2) and a similar estimated mean difference of −0.66 pp (95% CI, −1.14 to −0.18 pp; eTable 3 in Supplement 2). eFigure 2 in Supplement 2 displays estimated means at different time points for the largest site (site 3), and eFigure 5 in Supplement 2 displays the smooth estimates. No violations of assumptions of the PLMM were found.

**Table 2.** Primary Outcome for Patients Receiving Hemodialysis by Group

Primary outcome: interdialytic weight gain^a^	Mean (95% CI), %		Difference, pp	*P* value
	Intervention (n = 68)	Control (n = 71)		
At 0 d	3.74 (3.26 to 4.22)	3.44 (2.96 to 3.92)	0.30 (0.02 to 0.58)	.01^b^
At 90 d	3.58 (3.15 to 4.00)	3.53 (3.11 to 3.96)	0.05 (−0.12 to 0.21)	
At 180 d	3.42 (3.01 to 3.82)	3.62 (3.22 to 4.03)	−0.21 (−0.40 to −0.02)	
At 270 d	3.26 (2.83 to 3.68)	3.72 (3.30 to 4.14)	−0.46 (−0.78 to −0.14)	
Difference between 180 and 90 d	−0.16 (−0.29 to −0.03)	0.09 (−0.03 to 0.22)	−0.25 (−0.42 to −0.09)	NA
Difference between 270 and 90 d	−0.32 (−0.58 to −0.06)	0.19 (−0.06 to 0.43)	−0.51 (−0.84 to −0.17)	NA
Difference between 270 and 180 d	−0.16 (−0.29 to −0.03)	0.09 (−0.03 to 0.22)	−0.25 (−0.42 to −0.09)	NA

Abbreviations: NA, not applicable; pp, percentage point.

^a^ A piecewise linear mixed model adjusting for enrollment site with random intercepts for patients and random slopes for days has been used. The knot is at the start of the intervention period (at 90 days). The expected mean of the outcomes at the different days for the largest site have been obtained using appropriate contrasts based on the piecewise linear mixed model.

^b^ The *P* value corresponds to the likelihood ratio test of the interaction between time and intervention arm in the postintervention period.

### Secondary Outcomes

The rate per 30 days of shortened dialysis sessions was lower in the Navigate-Kidney group vs standard care between preintervention and postintervention periods (median [IQR], 0.1 [−1.2 to 1.1] vs 0.6 [−0.5 to 1.8]; *P* = .02). The rate per 30 days of missed dialysis sessions did not differ between the Navigate-Kidney group vs standard care (median [IQR], 0.0 [0.0-0.2] vs 0.0 [0.0-0.3]; *P* = .41). There was no difference in hospitalization, emergency department visits, systolic blood pressure, or serum phosphorus between groups (Table 3). eFigures 3 and 4 in Supplement 2 display the predicted means over time for phosphorus and systolic blood pressure at site 3 (the largest). With respect to patient-reported outcomes, there was a difference in the Patient Activation Measure of 4.30 (95% CI, 1.70-7.20) points, with median (IQR) scores of 1.8 (−2.2 to 5.5) points and −2.2 (−7.4 to 2.5) points in the intervention vs the standard care group, respectively (*P* = .005; Table 3). Also, there was a difference in patient activation level, with 38 participants (56%) and 30 participants (43%) in levels 3 (taking action) and 4 (maintaining behaviors and pushing further) in Navigate-Kidney vs standard care, respectively (*P* = .04; eTable 4 in Supplement 2). A difference of 5.21 (95% CI, 1.04-8.33) points was also observed in the summary score of the Kidney Disease Quality of Life instrument, with median (IQR) scores of 2.1 (−4.2 to 7.3) points and −2.1 (−9.1 to 3.1) points in Navigate-Kidney vs standard care, respectively (*P* = .008). No statistically significant differences were observed in other patient measures or in kidney treatment adherence attitudes. Social risks (Table 3) were generally similar; 10 participants (15%) in the Navigate-Kidney group reported taking lower doses of medication less often at follow-up than baseline to make the medicine last longer, compared to 1 participant (1%) in standard care (*P* = .02), and 17 participants (25%) in the Navigate-Kidney group reported talking to people who they cared about and felt close to more often at follow-up than baseline, compared to 7 participants (10%) in standard care (*P* = .03).

**Table 3.** Secondary Outcomes for Patients Receiving Hemodialysis by Group

Secondary outcome	Median (IQR)		Hodges-Lehmann estimation (95% CI)	Wilcoxon *P* value
	Intervention (n = 68)	Control (n = 71)		
**Hemodialysis adherence measures**				
Rate per 30 d of missed dialysis sessions	0.0 (0.0 to 0.2)	0.0 (0.0 to 0.3)	0.00 (−0.16 to 0.00)	.41
Rate per 30 d of shortened dialysis sessions	0.1 (−1.2 to 1.1)	0.6 (−0.5 to 1.8)	−0.69 (−1.32 to −0.10)	.02
Predialysis sitting systolic blood pressure, mean (95% CI), mm Hg				
At 0 d	151 (145 to 157)	151 (145 to 158)	−0.49 (−3.76 to 2.77)	.52
At 180 d	150 (145 to 156)	152 (146 to 157)	−1.52 (−4.46 to 1.41)	
Serum phosphorus, mean (95% CI), mg/dL				
At 0 d	5.81 (5.21 to 6.41)	5.95 (5.36 to 6.53)	−0.14 (−0.76 to 0.49)	.92
At 180 d	5.91 (5.45 to 6.37)	5.94 (5.49 to 6.39)	−0.03 (−0.39 to 0.32)	
**Change in baseline to follow-up in patient-centered assessments**				
Renal Adherence Attitudes Questionnaire	−0.5 (−5.2 to −5.0)	1.0 (−5.8 to 6.0)	−1.00 (−4.00 to 2.00)	.38
Patient Activation Measure	1.8 (−2.2 to 5.2)	−2.2 (−7.4 to 2.5)	4.30 (1.70 to 7.20)	.005
KDQOL Kidney summary score^a^	2.1 (−4.2 to 7.3)	−2.1 (−9.1 to 3.1)	5.21 (1.04 to 8.33)	.008
Burden of kidney disease	0.0 (−6.2 to 12.5)	0.0 (−12.5 to 10.9)	0.00 (−6.25 to 6.25)	.42
Symptoms/problems	0.0 (−6.2 to 6.2)	−2.1 (−8.3 to 4.2)	2.08 (0.00 to 6.25)	.09
Effects of kidney disease	3.1 (−6.2 to 9.4)	−3.1 (−15.6 to 6.2)	6.25 (0.00 to 12.50)	.02
KDQOL short form 12: physical health composite	1.7 (−3.4 to 6.1)	0.8 (−4.8 to 5.1)	1.53 (−1.16 to 4.42)	.26
KDQOL short form 12: mental health composite	−1.6 (−5.0 to 2.0)	−0.0 (−4.6 to 5.1)	−1.41 (−3.95 to 1.18)	.30
**Health care utilization**	**No. (%)**		**Odds ratio (95% CI)^b^**	***P* value^b^**
Any hospitalization				
Within 90 d prior to intervention	11 (16)	6 (8)	0.58 (0.11 to 2.84)	.50
Within 90 d after intervention	13 (19)	12 (17)		
Any emergency department visit				
Within 90 d prior to intervention	2 (3)	2 (3)	0.54 (0.02 to 13.02)	.99
Within 90 d after intervention	2 (3)	4 (6)		
**Social risks**				
Better housing situation at follow-up than baseline	6 (9)	7 (10)	0.86 (0.27 to 2.70)	.79
Less worried about losing housing at follow-up than baseline	10 (15)	6 (9)	1.81 (0.62 to 5.29)	.28
Took smaller doses of medicine less often at follow-up than baseline to make the medicine last longer	10 (15)	1 (1)	12.14 (1.51 to 97.77)	.02
Skipped doses less often at follow-up than baseline to make the medicine last longer	7 (11)	0	NA	NA
Spent less money on food, heat, or other basic needs less often at follow-up than baseline so there is money for medicine	7 (11)	8 (12)	0.90 (0.31 to 2.65)	.86
Has better transportation for receiving hemodialysis at follow-up than baseline	8 (12)	9 (13)	0.90 (0.33 to 2.5)	.85
Has better transportation for medical appointments or getting medications at follow-up than baseline	8 (12)	10 (14)	0.80 (0.30 to 2.17)	.66
Has better transportation for nonmedical meetings, appointments, work, or from getting needed things at follow-up than baseline	5 (7)	7 (10)	0.71 (0.22 to 2.37)	.58
Talk to people that you care about and feel close to more often at follow-up than baseline	17 (25)	7 (10)	2.95 (1.14 to 7.67)	.03

Abbreviations: KDQOL, Kidney Dialysis Quality of Life; NA, not applicable.

SI conversion factor: To covert serum phosphorus to mmol/L, multiply by 0.323.

^a^ Higher KDQOL scores mean better quality of life.

^b^ The *P* value and odds ratios are based on exact conditional logistic regression.

## Discussion

We demonstrated that a community-partnered and designed, CHW-led intervention modestly lowered IDWG, reduced shortened hemodialysis sessions, and improved patient activation for Hispanic and Latino participants with hemodialysis-dependent kidney failure. To our knowledge, this is the first clinical trial to show that a culturally and language-tailored intervention for Hispanic and Latino participants receiving in-center hemodialysis improves biomedical and patient-reported outcomes as well as healthy behaviors.

Providing Hispanic and Latino patients with consistent, culturally tailored support addresses both the social and psychological barriers that may limit adherence to dietary restrictions. Qualitative studies have found that Hispanic and Latino individuals with dialysis-dependent kidney failure feel isolated from their families, making it more challenging for them to feel supported emotionally with meal preparation and dietary restrictions and putting them at risk for diet-related complications, such as high IDWG.^2^ In Navigate-Kidney, the CHW acted as a cultural broker, meeting with the participant and the dialysis clinic dietitian. In some cases, the CHW shared with the dietitian important, otherwise unappreciated, details such as types of food in participant homes and information about who shopped and cooked for the participant. Explaining the connection between the salt content of food, thirst, water consumption, and subsequent IDWG helped participants understand why the dietary restrictions were relevant to their dialysis care and health. In addition, the CHWs used motivational interviewing to teach participants how to reduce IDWG through careful grocery shopping and cooking. Specifically, CHWs provided support with selecting less processed foods that were lower in salt and phosphorus and foods with lower carbohydrate and sugar content. In one case, a CHW helped a participant understand that adding chicken bouillon to cooking increased the salt content, driving thirst, and potentially increasing IDWG.

Compared with people in the general population, people receiving hemodialysis have 10- to 20-fold higher rates of cardiovascular morbidity and mortality. Chronic volume overload contributes to cardiovascular complications by worsening hypertension and myocardial strain and activates neurohumoral and inflammatory pathways, which contribute to arterial stiffness and left ventricular hypertrophy.^56,57,58^ A study of approximately 40 000 individuals with dialysis-dependent kidney failure showed that volume overload at hemodialysis initiation and persistent volume overload 1 year following hemodialysis initiation was strongly associated with death.^59^ Furthermore, attempts to remove excess volume during hemodialysis by ultrafiltration can also be detrimental, as higher rates of ultrafiltration are associated with multiorgan ischemia and higher rates of all-cause and cardiovascular mortality.^60^ The link between higher IDWG and higher ultrafiltration rates is particularly important, as ultrafiltration rates are one of the clinical metrics used to determine dialysis center reimbursement in the Centers for Medicare & Medicaid Services’ Quality Incentive Program.

We found that Navigate-Kidney not only reduced IDWG, but also improved patient activation, a key indicator of adherence that associates with favorable clinical, behavioral, and psychosocial outcomes.^61^ Specifically, we showed a 4-point difference in change in patient activation from preintervention to postintervention between the Navigate-Kidney and standard care groups. The Patient Activation Measure is a quality measure used by the Center for Medicare and Medicaid Innovation to rate clinicians enrolled in the Comprehensive Kidney Care Contracting models.^62^ The reduction in IDWG was modest, but prior data show a near-monotonic relationship between increasing IDWG and cardiovascular morbidity and mortality, suggesting that even modest decreases may have some effect on outcomes.^25,30^ Furthermore, the follow-up period was relatively short. Future research is needed to evaluate if the observed reduction in IDWG can be sustained and perhaps even improved as patients become more comfortable with the diet and lifestyle changes.

### Limitations

This trial has several limitations. The study was conducted in a single urban area in Colorado, which may limit generalizability. Since both control and intervention arms were implemented in the same clinic, contamination may have occurred. Some results for exploratory outcomes may be spurious due to the potential inflation of statistical significance caused by multiple testing. This trial was conducted during the COVID-19 pandemic, which created atypical clinical and social contexts and, in many cases, increased Hispanic and Latino participants’ social support needs. Hospitalizations and emergency department visits may have been missed due to inaccuracies or incompleteness in the electronic health record.

## Conclusions

In this randomized clinical trial, a culturally tailored CHW intervention modestly lowered IDWG and improved dialysis adherence and patient activation among Hispanic and Latino patients with hemodialysis-dependent kidney failure. In 2024, Medicare Part B implemented a reimbursement mechanism for community health integration, which includes CHW services. This opportunity for sustainable, reimbursable CHW services and the health benefits of CHW engagement demonstrated in this study suggest that providing CHW support for Hispanic and Latino individuals receiving hemodialysis is a practical and meaningful approach to improving patient outcomes.

## Notes

Supplement 1.
Trial Protocol

Supplement 2. **eFigure 1.** Navigate-Kidney Intervention for Individuals with Hemodialysis-Dependent Kidney Failure **eTable 1. **Study Measure Details
eReferences
**eTable 2.** Statistical Analysis Details **eTable 3.** IDWG Outcome for Patients Receiving Hemodialysis Randomly Assigned to Community Health Worker or Standard Arm, Assuming Different Pre-Intervention **eFigure 2.** Percent of interdialytic weight gain at the largest site (site 3) with 95% confidence limits, assuming same pre-intervention **eFigure 3. **Systolic blood pressure at the largest site (site 3) with 95% confidence limits, assuming same pre-intervention, along with smooth curves **eFigure 4. **Phosphorus at the largest site (site 3) with 95% confidence limits, assuming same pre-intervention, along with smooth curves **eTable 4. **Patient Activation Measure Level at Follow up for Patients Receiving Hemodialysis Randomly Assigned to Navigate-Kidney or Standard Care **eTable 5. **Characteristics of Study Population by Enrollment Site (n=139) **eTable 6. **Baseline Measures by Enrollment Site **eFigure 5.** Percent of interdialytic weight gain at the largest site (site 3) with 95% confidence limits with smooth curves, assuming same pre-intervention

Supplement 3.
Data Sharing Statement
