# Supplementary material for: Community Health Worker Support for Hispanic and Latino Individuals Receiving Hemodialysis: The Navigate-Kidney Randomized Clinical Trial
Source: JAMA Intern Med. 2025 Nov 7;186(1):56–66. doi: 10.1001/jamainternmed.2025.5305 (PMC12595546; doi:10.1001/jamainternmed.2025.5305)
Supplement: Supplement 1. — Trial Protocol [file jamainternmed-e255305-s001.pdf]

**Protocol #:**	20-0419
**Project Title:**	Peer Navigator study: Improving the well-being of Latinos on hemodialysis
**Principal Investigator:**	Lilia Cervantes, MD
**Institution:**	Denver Health and Hospital Authority
**Version Date:**	4/28/22

I. Hypotheses and Specific Aims

The goal of this project is to assess the feasibility and acceptability of a pilot RCT of a culturally tailored peer-navigator (**PN**) intervention to improve patient-centered and clinical outcomes for Latino patients with end-stage kidney disease **(ESKD**). We will compare a culturally tailored intervention that includes a PN to control (standard care). In the culturally tailored intervention, the bilingual PN will provide support with social challenges during 5 visits. We will assess the feasibility of (1) referral, (2) recruitment, (3) retention, (4) intervention implementation, and (5) data collection. We will also assess various outcomes including inter-dialytic weight gain and other adherence and patient-centered outcomes.

> **Specific Aim 1**: Conduct a pilot RCT of the peer navigator intervention to assess feasibility, acceptability, as well as outcomes of the proposed peer navigator intervention.
>
> **Hypothesis 1:** A culturally tailored intervention that consists of a bilingual/culture-concordant peer navigator that provides support with social challenges for Latino end-stage kidney disease (ESKD) patients, is feasible and acceptable.

I. Background and Significance

<u>Rationale for peer navigation in the ESKD setting.</u> Community Health Workers (i.e. Peer Navigators) have improved health outcomes within the Latino community, with increases in cancer screening rates, improving nutrition, and addressing mental health.(1-5) Community based participatory action theory is central to this approach and has been advocated as a model to reduce health disparities.(6) To our knowledge, there is little research on the use of peer navigator interventions to improve outcomes among Latinos with ESKD on dialysis. Patient navigation is well-suited for Hispanic ESKD patients for several important reasons. First, ESKD disproportionately affects Hispanics and other racial/ethnic minorities who also face burdens related to social challenges that altogether increase their risk of progression from CKD to ESKD and worse ESKD outcomes.(7-15) Some of the social challenges are remediable and may be addressed by a PN intervention. Low health literacy, as one example, is more common among low socio-economic status patients with ESKD and is associated with non-adherence to care, which is in turn, a risk factor for increased mortality.(16-18) For patients that experience low health literacy, a PN could help establish an environment that promotes health literacy by providing individualized education.

Second, persons with ESKD receive dialysis in their community by a healthcare workforce that does not reflect the diversity of the U.S. population.(19-21) Diversity in the healthcare workforce improves access to healthcare for underserved patients and improves racial and ethnic minority choice and satisfaction.(22-31) While efforts underway to increase diversity in the overall healthcare workforce progress slowly, PN programs in which the PN matches the diversity of the patient population, can serve as a bridge to the healthcare providers at the dialysis facility and can be implemented more immediately. Moreover, the diagnosis of ESKD is complex requiring a change in schedule and diet as well as clinical appointments for vascular access and kidney transplantation and having a culturally concordant PN provide emotional and social support can help patients overcome barriers. Third, dialysis facilities that predominantly serve minority and low-income patients tend to have worse performance on quality domains and may be more likely to receive a payment reduction.(7, 32-35) To promote high quality patient care in

the outpatient dialysis setting, the Centers for Medicaid and Medicare Services (CMS) implemented the ESKD quality incentive program, a value based purchasing model in which payments are linked to performance on quality care measures. In dialysis facilities that serve minority and low-income patients, PNs that are culturally and linguistically concordant may be particularly important to improve both quality and reimbursement. In summary, PNs can enhance the quality of care racial/ethnic minorities receive by providing support with social challenges and by serving as a bridge to the dialysis healthcare providers.

Current State of Science of Patient Navigation in Kidney Disease. To our knowledge, there are six PN published studies focused on patients with CKD. The primary outcomes described include change in eGFR(36), kidney transplantation process step completion(37, 38), increase in potential living kidney donor(39), increase wait-listing for kidney transplantation(40), and a feasibility study to improve health-related quality of life of dialysis patients.(41) The professional experience of the PN varied among the studies. Two employed lay PNs with college degrees(36, 41) and two employed PNs that were professional social workers.(39, 40) Culture and language concordance varied across the studies. The PNs in one study received cultural sensitivity training on the target African American community(39) and the PNs were culture and/or language concordant in two studies.(40, 41) Three studies employed PNs who were ‘peers’ (i.e. had prior personal experience with ESKD). In two studies, the PNs were previous transplant recipients(37, 38, 42) and in one study, the PN had previously cared for a family member with ESKD.(41) The experiences of peer PNs was further explored by the two studies that employed prior kidney transplant recipients. They found that because of the shared experience with CKD, the PNs felt a sense of personal satisfaction and enjoyed sharing in the success of dialysis patients.(42) Trust between the PN and patient was specifically described as critical to study success in three of the PN studies.(36, 39, 41) Altogether, we learn from the CKD PN programs that there is great variation in terms of PN experience and training.

Rationale for peer navigation focus on Latinos with ESKD on HD: Latinos represent 17% of the United States (U.S.) ESKD population yet remain under-represented in existing ESKD research.(43) Hispanic ethnicity and depressive affect are highly predictive of a reduced health-related quality of life (**HRQOL**) (44-46) and reduced renal therapy adherence (47-50). Reduced renal therapy adherence is associated with a higher morbidity (51-55) and higher mortality (45, 53, 56-64) among patients with ESKD. Multiple factors (e.g., symptom burden, mental health) associated with ESKD can affect renal therapy non-adherence. In Latinos, these concerns are compounded by cultural factors (i.e., behaviors and values), and social factors (i.e. , socioeconomic status, health literacy).(65-70). ***A peer navigator (PN) intervention that provides individualized support by addressing patient-identified barriers and facilitators to care within a dialysis center has the potential to improve renal therapy non-adherence.***

II. Preliminary Studies/Progress Report

Palliative care: Barriers and Needs in Latinos with ESKD (Harold Amos Medical Faculty Development Award from the Robert Wood Johnson Foundation Grant 2015212) COMIRB 14-0938

> **Specific Aim 1:** To identify unique needs for, and barriers to, palliative care for Latinos with ESKD using a mixed methods approach by administration of quantitative survey instruments followed by semi-structured interviews of patients, family caregivers, and healthcare providers.
>
> **Results:**

(1) **Qualitative results of Latino ESRD palliative care preferences:** We conducted 20 qualitative interviews of Latinos on hemodialysis between February and July 2015 and identified four themes: Avoiding harms of medications (fear of addiction and damage to bodies, effective distractions, reliance on traditional remedies, fatalism: the sense that one's illness is deserved punishment); barriers and facilitators to ACP: faith, family, and home (family group decision-making, family reluctance to have ACP conversations, flexible decision-making conversations at home with family, ACP conversations incorporating trust and linguistic congruency, family-first and faith-driven decisions); enhancing well-being day-to-day (supportive relationships, improved understanding of illness leads to adherence, recognizing new self-value, maintaining a positive outlook); and distressing aspects of living with their illness (dietary restriction is culturally isolating and challenging for families, logistic challenges and socioeconomic disadvantage compounded by health literacy and language barriers, required rapid adjustments to chronic illness, demanding dialysis schedule). These findings were published in CJASN in 2017(71)

(2) **Quantitative results of Latino ESRD palliative care preferences:** We surveyed 61 Latino ESRD participants on hemodialysis between September and December 2014 using a modified version of Davison's survey that explores palliative care preferences and needs (CITATION). We found that the majority of participants want to discuss their physical symptoms (89%) and quality of life (95%), be informed about prognosis (98%), and be prepared and plan ahead for end of life (90%), yet most reported no prior end-of life discussion with their nephrologist (84%) or family (71%). The majority of participants preferred to have end-of-life conversations early after dialysis therapy initiation but before becoming ill (85%), on a routine basis (87%), and during a dialysis session (48%) or at home (38%). We also found that Latinos rely on family and friends for social and emotional support (87%), choose family to make medical decisions (95%), and want to have family actively involved in medical decision making (93%). The majority (70%) would prefer resuscitation if their heart stopped and few (15%) regret the decision to start dialysis therapy. These findings were published in AJKD in 2016 (72).

Apoyo con Cariño: A Patient Navigator Intervention to Support Latinos on Dialysis (Harold Amos Medical Faculty Development Award from the Robert Wood Johnson Foundation Grant 2015212) COMIRB 14-0938

Specific Aim 1: Evaluate the feasibility of a peer navigator intervention for 40 Latinos on scheduled thrice weekly dialysis.

Specific Aim 2: Evaluate exploratory efficacy measures that will focus on quality of life, symptom burden, adjustment to illness, self-efficacy, and psychosocial adjustment to illness.

Results:

(1) **Intervention Development and Cultural Tailoring:** A community advisory panel composed of patient and caregiver stakeholders provided guidance on the tailoring

of the PN intervention, informed by our previous research on the palliative care preferences and needs of Latino ESRD patients. The PN intervention focused on four domains: (1) advance care planning (ACP); (2) care coordination; (3) dietary support; and (4) mental health support. We chose a maximum of five planned visits. The PN continued to be available to participants if they requested additional visits.

(2) **Feasibility Pilot Study of the Peer Navigator Intervention for Latino ESKD Patients on HD:** The pilot feasibility trial of 40 Latinos with ESRD allowed the research team to refine methods for the navigator training, recruitment, enrollment, tracking the intervention "dosing", and data collection methodology. Of 49 eligible patients, 40 (82%) agreed to participate. The majority of participants received scheduled outpatient HD (75%). No participants withdrew from the intervention. One participant died and with hospice. The mean (SD) number of PN visits per participant was 7 (2) and the mean (SD) length of the visits was 97 minutes (49). The majority of visits took place at the hemodialysis facility (59%) and home (27%). The vast majority of participants reported that the PN improved their quality of life as a patient on HD (95%). The intervention achieved feasibility goals and was well received by participants. The results of our study were published in the Journal of Palliative Medicine in 2019 (41).

III. Research Methods

A. Outcome Measure(s)

An overview of Specific Aim 1 outcomes and measures are listed in **Table 1** & details are in **Appendix A.** We will assess feasibility, acceptability, as well as patient-centered and clinical outcomes.

Table 1. Outcomes

	Domain	Outcome measure and details	Timeline of data collection
Outcomes	Renal Therapy Adherence	(1) Interdialytic weight gain	Data collection 3 months prior to consent (collected retrospectively) and 6 months following study completion
		(1) Hemodialysis adherence: Missed and shortened (>10 minutes)	Hemodialysis attendance assessed monthly. Data collection 3 months prior to consent (collected retrospectively) and 6 months following study completion.
		(2) Albumin, potassium and Phosphorus.	Albumin & Phosphorus assessed monthly. Data collection 3 months prior to consent (collected retrospectively) and 6 months following study completion.
		(3) Interdialytic Weight Gain (73-75)	IDWG assessed weekly from medical chart. Data collection 3 months prior to consent (collected retrospectively) and 6 months following intervention completion.

		(4) Composite index of adherence: IDWG, pre dialysis potassium blood level, phosphorus level, and missed hemodialysis session	Data collection 3 months prior to consent (collected retrospectively) and 6 months following intervention completion.
		(5) Kidney Transplantation: interest in pursuing, placement on list, receipt of kidney transplantation.	At study completion (yes/no) & at 6 months following intervention completion
	Other outcomes	(6) ED visits and hospitalizations.	ED visits and hospitalizations from medical chart. Data collection 3 months prior to consent (collected retrospectively) and 6 months following study completion.
		(7) Social determinants of health composite survey	Collected at time of consent and study completion
		(8) What is the most pressing social challenge?	Collected during first patient visit (in first PN visit note) and then at time of completion (in last PN visit note)
		(9) Social Isolation (PROMIS measures)	Collected at time of consent and study completion
		(10)Patient Activation Measure (PAM-13)	Collected at time of consent and study completion
		(11) Self-Efficacy (PROMIS measures)	Collected at time of consent and study completion
		(12) KDQOL SF-36	Collected at time of consent and study completion
		(13) Renal Adherence Attitudes Questionnaire	Collected at time of consent and study completion
Acceptability		1) Qualitative interviews with 8 interdisciplinary clinicians, two peer navigators, and 50 patients.	Collected at study completion

B. Description of Population to be Enrolled

Total participants, **n=160**

Inclusion Criteria:
(1) Self-identify as Latino
(2) Age between 18 and 90 years
(3) Diagnosed with end-stage kidney disease
(4) Received standard (thrice-weekly HD) for at least 3 months
(5) No active substance use (e.g., heavy etoh or opiates)
(6) Speak English or Spanish as a primary language
(7) Participants must be able to provide informed consent

Exclusion Criteria:
(1) Active suicidal intent
(2) Present or past psychosis or bipolar disorder
(3) Patient to receive kidney transplantation in the next 3 months

Description of criteria for withdrawal from study:
(1) Participant withdrawal
(2) PI termination

Research Sites: This study will recruit participants from Fresenius dialysis facilities. The dialysis facilities within each of these large dialysis organizations are located in Denver and have a high proportion of Latino/na ESKD HD patients.

Fresenius dialysis facilities:
(1) Fresenius Kidney Care East Denver – Michel Chonchol (Co-I and mentor) is Medical Director
(2) Fresenius Kidney Care Rocky Mountain – Michel Chonchol (Co-I and mentor) is Medical Director
(3) Fresenius Kidney Care Pavilion Dialysis – Seth Furgeson (Co-I) is Medical Director
(4) Fresenius Kidney Care Sloans Lake – Seth Furgeson (Co-I) is Medical Director

C. Study Design and Research Methods

This is a multi-clinic, longitudinal, mixed methods (qualitative & quantitative), observational and developmental study.

Recruitment: At each clinical site, the Medical Director (also Co-I on this study) will be the study's onsite coordinator. The peer navigator (PN) will work with the medical director to identify patients who may be potentially eligible for this study (HIPAA A or waiver are not required to identify eligible patients to be approached for recruitment, because co-investigators have a clinical relationship with the patients. Their relationship extends to all members of the study team for HIPAA research purposes). Patient level data that will be accessed for study screening purposes include: medical diagnoses and time on dialysis. The medical director, who knows the patients well given his/her clinical contact with the patients, will identify eligible patients.

If patients meet the eligibility criteria, the medical director (onsite coordinator) will provide patient contact information to the PN who will then contact the patient. The PN will use a recruitment script (**Appendix B).** The consent process will be in person. If patients

agree to participate, they must give informed consent separately in writing or electronically with the REDCap e-Consent feature. The patient must also agree with the HIPAA language in the consent form. These forms will be provided in English and/or Spanish. The PN will read and summarize each section of the consent form, assessing comprehension as well as willingness to consent. The consent form includes a description of the purpose of the research, risks and benefits, description of alternatives to participation (regular standard of care) and contact information for the PI/Co-I and the human participants' protection committee (IRB).

With support from her research mentors, the PI will oversee the recruitment and consent process.

Possible benefits of the study: Potential benefits include receiving additional support with social challenges from the Patient Navigator. This study is designed for the researcher to learn more about how to improve the well-being and care for Latino/Latina patients with end-stage kidney disease receiving hemodialysis.

Retention: We demonstrated excellent retention with our PN feasibility study (see preliminary results). In order to retain participants, we will: (1) provide full verbal and written descriptions of the study requirements to potential participants prior to signing the informed consent form on paper or electronically; (2) address all participant questions/concerns in detail during the consent process; (3) explain the importance of the research being performed; (4) maintain regular (at least weekly) communication with study participants through in-person check-in visits and phone calls; (5) perform the study in the dialysis facility where the participant receives thrice weekly hemodialysis; and (6) respond to all participant inquiries in a timely manner.

Participant Compensation: We will provide patients with $60 to compensate them for their time at the consent visit, $20 during each additional visit, and then $60 compensation following the post-intervention measurement visit. In total, participants will receive $220.

Baseline Assessment: Following consent and prior to randomization, participants will have an initial assessment that will include collection of baseline sociodemographic information (e.g., gender, age, comorbidities to assess the Charlson Comorbidity Index, see **Appendix A** for details) and baseline measures (**Table 1 and details in Appendix A**). The PN will strive to build trust and rapport with the patient during the consent process so that the patient feels more comfortable answering measures. Sociodemographic information will include contact information (phone, address, and email), date of birth, place of birth, if foreign born then number of years in the US, ethnicity, primary language, and socioeconomic status (employment, average annual income, highest education level achieved, and home ownership). The details of sociodemographic information to be collected is in Appendix A. The baseline measures (**Table 1 & Appendix A**) Social Determinants of Health (composite survey), Social Isolation (PROMIS measures), Patient Activation Measure (PAM-13), Self-Efficacy (PROMIS measures), and the KDQOL SF-36 (see **Appendix A** for details). The baseline measures take approximately 40-60 min to complete. The PI will review medical records for detailed medical data on diagnoses and comorbidities (to calculate a Charlson Comorbidity Index and calculate dialysis vintage), and for a list of current medications and dosages, as well as the renal therapy adherence data.

Final Assessment: Once the participant has completed the intervention, a research assistant (that has never met the patient) will assess satisfaction with using semi-structured qualitative interview. The PI will conduct a chart review to collect the outcomes data for 6 months following intervention completion.

Randomization: The co-investigator (ZY) will prepare blocked randomization (random permuted blocks) for assigning participants to intervention or control group within each clinic site to avoid serious imbalance in the number of subjects between the two groups throughout the study period. The block size also will be randomly varied between 2, 4, and 6 to preserve the randomization scheme.

Intervention: Following consent and completion of baseline measures, the PN will schedule the first visit within 1-2 weeks. The function of the initial visit is to establish trust and ensure a more personal approach with participants. The community-based PN intervention is grounded in core Latino values (e.g. trust, personalized relationships). The core elements of the PN intervention include patient motivational interviewing as well as patient activation, empowerment (e.g., help with scheduling of healthcare appointments and re-scheduling of missed HD sessions), education (e.g., education of ESKD and need for renal replacement therapy), and social challenges (e.g., access to resources for transportation, benefits, immigration issues). The duration, individuals present during the visit, and content discussed will be documented in the visit form (**Appendix C**).

Intervention timeline: The first PN visit will take place within 1-2 weeks of consent (Figure 1). Each of the 5 subsequent PN visits will take place every 1-2 weeks. We expect patients to complete the intervention within 2-3 months of consent. The study completion measures will be collected by the research assistant within 1-2 weeks of study completion.

Figure 1. Proposed Timing of Intervention Encounter

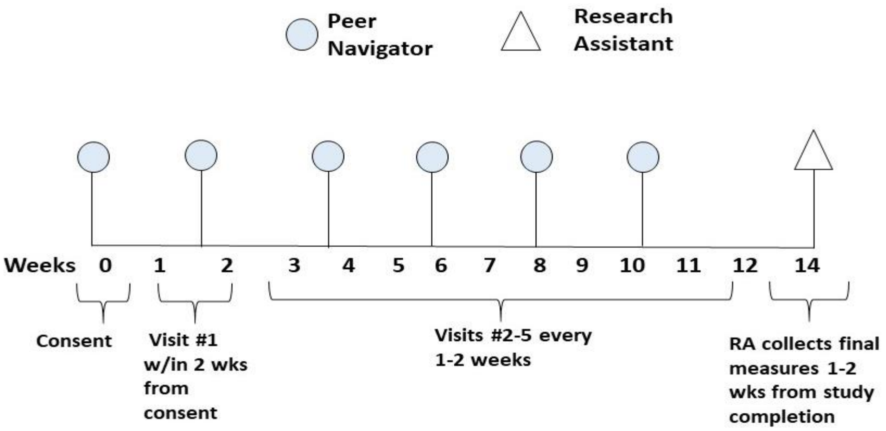

PN Visit Content: The PN will arrange to meet with participants during HD or at home or other preferred location. During the first study visit, the PN will provide and review the educational materials that cover the domains (**Table 2**), understand social challenges faced by patient, and use motivational interviewing to establish rapport and empathize with patients regarding difficult to change adherence behavior through listening. Additionally, during that visit, the PN will work with the participant to create a plan for subsequent visits based on the participant's needs. Participants will be at different

points in their illness trajectory and their acceptance of the domains in our intervention will vary. The PN visits will thus vary in both content and activities covered. The delivery of the intervention including its content and number of visits will be customized to the needs of each participant and documented in the visit form (**Appendix C).**

Table 2. Framework of PN Visit Content

Adherence	Social Challenges
• Review educational material (from NKF) re ESKD/HD and how this ties to diet<br><br>• Motivational interviewing (MI): (1) Establish rapport and empathize with patients regarding difficult to change behavior through listening. (2) Help patients develop connection between current behavior and goals/values that are important to the patient (e.g. adherence renal therapy such as low salt renal diet and fluid). (3) At each visit, help patient develop a connection between current behavior and values/goals as well as help patient develop discrepancy between behavior and goals/values. (4) Support autonomy and self-management.<br><br><u>Examples</u>: If patient is non-adherent to IDWG, MI to discuss low sodium diet/ fluid intake goals. If a dialysis session is missed, MI to dialysis adherence goals, re-schedule dialysis and prevent future missed dialysis.<br><br>• During each visit, review education materials and discuss goals related to dialysis and diet adherence.	• Discuss concerns related to health, finances, housing, transportation, and family.<br><br><u>Example:</u> If trouble with transportation lead to missed dialysis, re-schedule dialysis & troubleshoot transportation issues.<br><br>• Support high quality communication. Provide Spanish language interpretation while at the dialysis chairside when healthcare provider meets with patient.

Navigator or Patient Initiated Visits: A maximum of 5 visits was chosen based on our findings from the 1-arm feasibility PN study described in our preliminary research section above. The navigator will continue to be available to the participant and family as needed to facilitate adherence and overcome social challenges throughout the study time period. After the intervention is complete, the navigator will arrange additional visits if requested by the participant or family during the study period.

Navigator integration to dialysis facility interdisciplinary team: The PN will attend the Plan of Caremeeting for those participants in the intervention arm. Separately, if a participant in the intervention arm misses hemodialysis, the LCSW can notify the PN. The PN can reach out to patients and document why dialysis was missed and assess if patient needs assistance with re-scheduling dialysis.

Control group: Control group participants will receive the same educational materials that are provided to the intervention group in the appropriate language.

D. Description, Risks and Justification of Procedures and Data Collection Tools

Research tracking chart & fidelity to the intervention: A study tracking chart will be assigned to each subject. Each patient consent (if signed on paper) and a hard copy of the study tracking chart will be stored in a locked file cabinet in the PI's (LC) office in a secured office in the secured Leprino office building on the University of Colorado Anschutz Medical campus. The PN will transfer this information to a standardized electronic form in RedCap.

The content of each visit in the study tracking chart includes the duration of the visit, other individuals present during the visit, and content discussed (**see Appendix C**). With respect to the content, the navigator will record details about the topics discussed; specifically, there will be a section for social challenges/barriers faced by patient and how those are being addressed as well as a section for adherence in which the PN will describe the patient's goals and steps taken to meet those goals. There will be an accurate count of the total number of visits each participant received from the navigator. In addition to the in-person visits, the navigator will also track the number, duration, and content of phone calls.

The dose will be tracked carefully and this will allow for future evaluation of the fidelity to the intervention necessary to achieve the outcome goals and to increase reproducibility. If the participant refuses navigator visits, the navigator will attempt to contact the participant to better understand the reason for wishing to withdraw from the study. The reason for drop out will be written in the participant's tracking chart.

Data Collection and Management: The PI created a data collection manual with detailed description of each measure with instruction for its accurate collection. The manual will have space to record any questions that arise during data collection (**see Appendix A**).

Baseline: After consent, the PN will collect baseline demographic information as well as baseline measures (see **Table 1 & Appendix A)**. All quantitative data will be entered into REDCap, a secure, HIPAA compliant, web-based application for building and managing online databases and is provided free of charge bythe University of Colorado . The data will be exported without patient identifiers. The data will be entered into REDCap by the patient navigator. Participants will be assigned a unique non-linked identifier code. The link between the identifier code and patient identifiers will be stored separately from research data on the University of Colorado firewalled, password protected, virtual private network on a server accessible only to study team members. Shared files may be stored on a HIPAA compliant OneDrive folder in order for all approved study personnel to have access. Once all participants have completed the study, files will be transferred from the shared OneDrive folder to the secure network folders during analysis.

Final Assessment: Once the participant has completed the intervention, the research assistant will assess satisfaction with care using a qualitative semi-structured interview (interview guide in **Appendix A)** as well as complete the study completion measures described in **Table 1 & Appendix A**. The PI will conduct a chart review to collect the outcomes data for 6 months following study completion for each participant. All quantitative data will be entered into REDCap, a secure, HIPAA compliant, web-based application for building and managing online databases and is provided free of charge by University of Colorado. The data will be exported without patient identifiers. The data

will be entered into REDCap by the patient navigator. Participants will be assigned a unique non-linked identifier code. The link between the identifier code and patient identifiers will be stored separately from research data on the University of Colorado firewalled, password protected, virtual private network on a server accessible only to study team members

Chart Review: The PI (Lilia Cervantes) will review medical records to obtain adherence data (e.g., missed hemodialysis sessions, inter-dialytic weight gain, phosphorus, potassium, kt/v, albumin) and co-morbidity measures for the Charlson Comorbidity Score. The Charlson Comorbidity score will include collection of the following variables: age, albumin, comorbidities (e.g., liver disease, heart failure, lung disease, connective tissue disease, dementia, cerebrovascular disease, peripheral vascular disease, diabetes, cancer, or AIDS). All quantitative data will be entered into REDCap, a secure, HIPAA compliant, web-based application for building and managing online databases and is provided free of charge by the University of Colorado. The data will be exported without patient identifiers. The data will be entered into REDCap by the patient navigator and /or the research assistant. Participants will be assigned a unique non-linked identifier code. The link between the identifier code and patient identifiers will be stored separately from research data on the University of Colorado firewalled, password protected, virtual private network on a server accessible only to study team members.

Sources of Materials: Data will be gathered from participants and from the participant's medical record.

Potential risks: Potential risks to participants are minimal. There are no foreseeable physical, social, psychological, or legal risks beyond those of participating in health-related research in general. One potential risk is loss of confidentiality. All written and audio-recorded data and consent materials (if consent was signed on paper) will be kept in a locked cabinet in a locked office that is in a building that is secured at all times. Audio-recordings will not be done in the Fresenius clinics or by phone when patient is at the Clinic to avoid other patient or patient PHI maybe picked up on the recording. All electronic materials will be kept in our REDCap database as described above.

Protection Against Risks: All efforts will be made to protect confidentiality of study subjects. All electronic data will be stored in REDCap, a secure, HIPAA compliant, web-based application database. All written and recorded data will be stored in a locked file cabinet in a locked office in a locked office suite in a building that is secured by electronic entry card during off business hours. There will be no questions regarding a participant's immigration status (whether documented or undocumented). Therefore, there is no significant risk to participants and the protection against risks is adequate. Provider-or subject- initiated complaints and adverse events will be monitored and reported to the appropriate regulatory agencies, including the IRB and NIH within the appropriate time period as specified by IRB-approved study procedures. If unanticipated incidental findings were to occur, the study PI will be responsible for reviewing these findings and ensuring that subjects receive appropriate clinical follow-up for these issues.

Data Safety and Monitoring Plan: Given the minimal risk posed to subjects who enroll in our study and the low complexity of the intervention, the study will be monitored by the PI, Dr. Cervantes in addition to the Colorado Multiple Institutional Review Board (COMIRB). Dr. Cervantes will be responsible for the ongoing oversight, review, and

reporting of adverse events related to the study. Dr. Cervantes will personally review each potential adverse event to determine the course of action, including reporting to appropriate groups, office, or agencies, or identifying a need for making protocol changes. Drs. Michel Chonchol, Romana Hasnain-Wynia, and John Steiner have extensive experience with the oversight of trials. During weekly mentorship meetings, my co-mentors will provide mentorship and guidance on any issues related to subject safety and retention.

Adverse events and Serious Adverse Events: Adverse and serious adverse events will be defined based on guidance from Colorado Multiple Institutional Review Board and the NIH. An Adverse Event (AE) is any unfavorable and unintended sign (including abnormal laboratory finding), symptom, or disease temporally associated with the use of a medical treatment or procedure, regardless of whether it is considered related to the medical treatment or procedure (attribution of unrelated, unlikely, possible, probable or definite). A serious adverse event (SAE) includes any untoward medical occurrence that at any dose results in death or the immediate risk of death, hospitalization or prolonging of an existing hospitalization, persistent or significant disability/incapacity or a congenital anomaly/birth defect. Research staff will alert Dr. Cervantes to any potential AE or SAE, who will assist with arranging any needed services for the participant. AE reporting will follow COMIRB policy – within 5 days of PI becoming aware of an AE as an unanticipated problem, a UAP Form will be submitted to COMIRB. If the AE does not meet the definitions of a UAP, an aggregated summary of AEs will be submitted at the next continuing review. AE reporting will also comply with any applicable institutional policies. In all cases, the PI, under advice of COMIRB, will determine what further, assessment, follow-up or action is required for an AE or SAE. A summary of SAEs that occurred during the study year will be part of the annual progress report that is submitted to both COMIRB and NIH.

Safety Monitoring: Given the minimal risk posed to subjects who enroll in our study and the low complexity of the intervention, the study will be monitored by the PI, Dr. Cervantes in addition to COMIRB. Dr. Cervantes is a fully licensed and board-certified adult internal medicine physician, and she provides care for patients with end-stage kidney disease. Dr. Cervantes is fully equipped to assess and respond to health-related concerns that arise due to study procedures. If a patient reveals intent to harm self or others, we will notify dialysis staff and the dialysis unit medical director. All patients will be at their dialysis center and the emergency protocol of the dialysis center will be followed if it is determined to be a true psychiatric emergency.

Complaints initiated by a patient or provider about the study procedures will be reported to the study PI (Dr. Cervantes) within 48 hours of the complaint being filed. All concerns will be addressed in a timely fashion by the PI who will also report those that constitute a possible adverse event or unexpected problem to COMIRB.

The dialysis center staff and the medical director of the dialysis center will be notified of the results of the PHQ-9 (depression questions). All hemodialysis centers in the U.S. are mandated by the Centers for Medicare and Medicaid Services End-stage renal disease Quality Incentive Program to do a yearly depression screen and when a patient is depressed, they must have documentation of a follow-up plan. It will be up to the patient's nephrology physician to document a follow-up plan with the patient per the usual standards of care in the dialysis center. All adverse events, serious adverse events, and unexpected problems that occur during the period of study enrollment or as

a result of participation in the study will be reported to COMIRB and the appropriate funding agency.

E. Data Analysis Plan

Effectiveness of Randomization: The co-investigator (ZY) will prepare the blocked randomization (random permuted blocks) for assigning participants to intervention or control group within each clinic site to avoid serious imbalance in the number of subjects between the two groups throughout the study period. Block randomization will apply and will base sex, age (within 2 years) and will be within clinic/facility.

Analysis of Outcomes: With pilot nature, this study is a randomized controlled trial of two parallel groups (treatment and control) designed to examine the effect of peer navigation on adherence to the standard prescription of hemodialysis for patients with end stage of kidney disease. An outcome used to power the clinical trial is the change from baseline to end of study in interdialytic weight gain (IDWG). The two-sample t-test is applicable in the comparison of the change between treatment and control to examine the treatment effect and thus is the basis for power analysis. Based on our experience, we are able to recruit around 141 patients into our study (about 2 patients per week for 80 weeks) and data of at least 128 patients will be available in final analysis (i.e., 10% dropout rate, 141(1-0.1) = 128). Assume that we will have equal number of patients (or almost equal) in the final analysis, then the power to detect an effect of 0.50 will be 80% with a two-sided type I error rate of 0.05. Based on report by Cukor and colleagues (2014), the standard deviation of the change in IDWG can be estimated as 0.3. This implies that the detectable difference in the change (of IDWG) between treatment and control is 0.5 x 0.3% = 0.15% (%Δkg per day), with our sample size and power of 80% and two-sided significance level of 0.05. Assume the average determined dry weight is 65 kg, then the average detectable difference of change in weight (kg) is about 65 x 0.15% = 0.10 kg.

For secondary outcomes of ED visit and hospitalization after missing a dialysis session, repeated measures analysis of binary data apply and the power will depend on the number of patients who miss a session during the study, the number of missed dialysis sessions, and the risk of an ED visit and hospitalization after missing a dialysis session. For a continuous secondary outcome such as the change from baseline to end of study in albumin, potassium, phosphorus, weight, and kt/v, the two-sample t-test is applicable and the expected total sample size of 141 will provide 80% power to detect an effect size of 0.50 after taking into account for 10% dropout rate.

A total of 141 patients are expected to be available in this study and will be equally randomized to the intervention and control groups, and block randomization by sex and age (within 2 years) will apply. Descriptive statistic will be provided for all variables. For example, mean and standard deviation will be calculated for a continuous variable and proportion for categorical and ordinal variables. The distribution of continuous variables will be checked and data transformation will be performed if appropriate. Median and quartile range will be calculated for a continuous variable if it is needed. Table and plot will be employed to present results.

Treatment effect evaluation will be based on the intention-to-treat principle. A two-sided significance level of 0.05 will be used in making conclusions. Analysis of covariance (ANCOVA) will be used to assess the treatment effect on IDWG and IDWG at end of

main intervention will be regressed on treatment group with adjustment for baseline IDWG. We will further adjust for sex and age. This analysis approach is applicable to other continuous outcomes. Furthermore, the number of missed dialysis sessions and the time to a missed dialysis session will be analyzed using the Poisson regression model and survival analysis of recurrent events to assess the intervention effect. For continuous outcomes measured multiple times, e.g. albumin, phosphorus, repeated measures data analysis with mixed effects models will be performed and the change from baseline to end of study will be compared based on the mixed model to assess the treatment effect. Similarly, adjustment for baseline covariates will be performed. We do not see reasons that any missingness of data is associated with any of the outcomes and thus we assumed missing at random for all potential missing data. Furthermore, repeated measures analysis will include all patients with a measurement in an outcome variable, which can potentially provide a higher power. All analyses will be performed by using SAS 9.4 or higher (SAS Institute, Cary, NC).

Rates of Refusal and Withdrawal: Given our preliminary research findings. We expect a low rate of refusal given our acceptance of 82% and a low withdrawal rate given our withdrawal rate of 0. We will conservatively plan for a refusal of 30% and a withdrawal rate of 10%. If we enroll 128 patients, using our conservative refusal and withdrawal rate, we will need to approach 170-180 patients.

Missing Data: Participants are scheduled for thrice-weekly outpatient hemodialysis at the dialysis facilities and for this reason, we do not expect missing data. Each dialysis facility collects hemodialysis adherence data as required by Federal CMS. Each dialysis facility is also required to document when a patient is hospitalized or seen in an emergency department. This is consistent with our experience in conducting the 1-arm pilot feasibility trail described in the preliminary research section.

Statistical Power: Based on the reports from Wileman (2016), Sharp (2005), Christensen (2002), and Cukor (2014), both the mean change in IDWG and the standard deviation of the change varied substantially under a treatment and a control (mean change varies from 0.18 to 1.3, standard deviation varies from to 0.3 to 1.0), but the estimated effect sizes varied in a narrow range from 0.5 to 0.7. We decided to conservatively target at a smaller effect size of 0.50. Further calculation indicated that, to detect an effect size of 0.50 with power 80% and a two-sided type I error rate of 0.05, we need to enroll a total of 128 patients with n= 64 per group. Finally, to take into account for a dropout rate of 20%, the total number of patients we need to enroll is 128/ (1-10%) = 141, which is the total number of the expected available patients. The power would be higher if more patients are actually available and/or dropout rate is lower.

For secondary outcomes of ED visit and hospitalization after missing a dialysis session, repeated measures analysis of binary data apply and the power will depend on the number of patients who miss a session during the study, the number of missed dialysis sessions, and the risk of an ED visit and hospitalization after missing a dialysis session. For a continuous secondary outcome such as the change from baseline to end of study in albumin, phosphorus, weight, and depressive score, the two-sample t-test is applicable and the expected total sample size of 141 will provide 80% power to detect an effect size of 0.50 after taking into account for 10% dropout rate.

Study Timeline: Each patient will stay in the study for one year. For all participants (regardless of randomization to control or intervention), we will collect data on adherence

for 3 months preceding enrollment and 6 months following completion of the study. For a patient randomized to the intervention group, intervention delivery will take approximately 2-3 months. The entire study duration from patient enrollment and data collection will be 3 years. Of the 3 years, all patients will be enrolled in the first 2.5 years (30 months) and the patients enrolled at 2 years of the study will complete follow-up at 3 years. The data and patient information will be securely stored until analysis is complete. Patient information such as paper consents will be stored for 7 years after that time the paper records will be destroyed. Patient's recordings will be permanently deleted from the secured PHI protected University of Colorado T-drive server. There are no future anticipated uses of the data at this time. Based on our experience, 2 or 2.5 patients will be enrolled per week in average and thus a total number of 160 to 200 patients are expected to be enrolled and equally randomized to the intervention and control group.

IV. Potential Scientific Problems

We may encounter difficulties with recruitment. If we encounter difficulties, we will have access to additional dialysis centers with a high proportion of Latino patients with end-stage kidney disease. Additionally, Dr. Chonchol (primary mentor) will support me in navigating the approval process for additional dialysis centers. Another challenge to consider is patient hospitalization. If a patient is hospitalized for < 2 weeks, they may resume the study intervention; however, if its > 2 weeks, they will start the intervention anew. Another limitation is my modest sample size. I will be able to detect trends in efficacy; however, a future R01 is necessary to power for efficacy.

V. Summarize Knowledge to be Gained

The overall goal of our project is to improve person-centered and clinical outcomes for Latinos with end-stage kidney disease on dialysis by providing them with culturally and linguistically tailored support from a peer navigator. This is a successful community-based model of peer navigation to deliver an intervention that will provide support with social challenges and adherence. Latinos represent 17% of the US community with end-stage kidney disease and compared to non-Latino whites, they are disproportionately burdened with social challenges including lower level education, income, and health literacy and they are more likely to report housing and food insecurity. Due to the unique cultural preferences and values of Latino patients on dialysis, our innovative and culturally tailored study has the potential to improve person-centered and clinical outcomes thereby reducing health disparities.

VI. References

1. Marlow NM, Simpson KN, Kazley AS, Balliet WE, Chavin KD, Baliga PK. Variations in coping stages for individuals with chronic kidney disease: Results from an exploratory study with patient navigators. Journal of health psychology. 2014.
2. Fischer SM, Cervantes L, Fink RM, Kutner JS. Apoyo con Carino: A Pilot Randomized Controlled Trial of a Patient Navigator Intervention to Improve Palliative Care Outcomes for Latinos With Serious Illness. Journal of pain and symptom management. 2014.
3. Freeman HP. Patient navigation as a targeted intervention: For patients at high risk for delays in cancer care. Cancer. 2015;121(22):3930-2.
4. Freeman HP. The origin, evolution, and principles of patient navigation. Cancer epidemiology, biomarkers & prevention : a publication of the American Association for Cancer Research, cosponsored by the American Society of Preventive Oncology. 2012;21(10):1614-7.
5. Meade CD, Wells KJ, Arevalo M, Calcano ER, Rivera M, Sarmiento Y, et al. Lay navigator model for impacting cancer health disparities. Journal of cancer education : the official journal of the American Association for Cancer Education. 2014;29(3):449-57.
6. Freeman HP. Patient navigation: a community centered approach to reducing cancer mortality. Journal of cancer education : the official journal of the American Association for Cancer Education. 2006;21(1 Suppl):S11-4.
7. Rodriguez RA, Sen S, Mehta K, Moody-Ayers S, Bacchetti P, O'Hare AM. Geography matters: relationships among urban residential segregation, dialysis facilities, and patient outcomes. Annals of internal medicine. 2007;146(7):493-501.
8. Crews DC, Gutierrez OM, Fedewa SA, Luthi JC, Shoham D, Judd SE, et al. Low income, community poverty and risk of end stage renal disease. BMC Nephrol. 2014;15:192.(doi):10.1186/471-2369-15-192.
9. Young BA, Katz R, Boulware LE, Kestenbaum B, de Boer IH, Wang W, et al. Risk Factors for Rapid Kidney Function Decline Among African Americans: The Jackson Heart Study (JHS). Am J Kidney Dis. 2016;68(2):229-39.
10. Jurkovitz CT, Li S, Norris KC, Saab G, Bomback AS, Whaley-Connell AT, et al. Association between lack of health insurance and risk of death and ESRD: results from the Kidney Early Evaluation Program (KEEP). Am J Kidney Dis. 2013;61(4 Suppl 2):S24-32.
11. Crews DC, Charles RF, Evans MK, Zonderman AB, Powe NR. Poverty, race, and CKD in a racially and socioeconomically diverse urban population. Am J Kidney Dis. 2010;55(6):992-1000. doi: 10.53/j.ajkd.2009.12.032. Epub 10 Mar 6.
12. Johns TS, Estrella MM, Crews DC, Appel LJ, Anderson CA, Ephraim PL, et al. Neighborhood socioeconomic status, race, and mortality in young adult dialysis patients. J Am Soc Nephrol. 2014;25(11):2649-57. doi: 10.1681/ASN.2013111207. Epub 2014 Jun 12.
13. Volkova N, McClellan W, Klein M, Flanders D, Kleinbaum D, Soucie JM, et al. Neighborhood poverty and racial differences in ESRD incidence. Journal of the American Society of Nephrology : JASN. 2008;19(2):356-64.
14. Tarver-Carr ME, Powe NR, Eberhardt MS, LaVeist TA, Kington RS, Coresh J, et al. Excess risk of chronic kidney disease among African-American versus white subjects in the United States: a population-based study of potential explanatory factors. Journal of the American Society of Nephrology : JASN. 2002;13(9):2363-70.
15. Crews DC, Kuczmarski MF, Grubbs V, Hedgeman E, Shahinian VB, Evans MK, et al. Effect of food insecurity on chronic kidney disease in lower-income Americans. Am J Nephrol. 2014;39(1):27-35.
16. Green JA, Mor MK, Shields AM, Sevick MA, Arnold RM, Palevsky PM, et al. Associations of health literacy with dialysis adherence and health resource utilization in patients receiving maintenance hemodialysis. Am J Kidney Dis. 2013;62(1):73-80. doi: 10.1053/j.ajkd.2012.12.014. Epub 3 Jan 24.

17. Green JA, Mor MK, Shields AM, Sevick MA, Palevsky PM, Fine MJ, et al. Prevalence and demographic and clinical associations of health literacy in patients on maintenance hemodialysis. Clin J Am Soc Nephrol. 2011;6(6):1354-60.
18. Kimmel PL, Peterson RA, Weihs KL, Simmens SJ, Alleyne S, Cruz I, et al. Psychosocial factors, behavioral compliance and survival in urban hemodialysis patients. Kidney Int. 1998;54(1):245-54. doi: 10.1046/j.523-755.998.00989.x.
19. United State Census Bureau. QuickFacts. United States. Table. 2018 [Available from: https://www.census.gov/quickfacts/fact/table/US/PST045218.
20. United States Census Bureau: Hispanic Heritage Month [Available from: https://www.census.gov/content/dam/Census/library/visualizations/2018/comm/hispanic-fff-2018.pdf.
21. Association of American Medical Colleges. Table A-8 Applicants to U.S. Medical Schools by Selected Combinations of Race/Ethnicity and Sex, 2015-16 through 2018-2019. [Available from: https://www.aamc.org/download/321472/data/factstablea8.pdf.
22. Komaromy M, Grumbach K, Drake M, Vranizan K, Lurie N, Keane D, et al. The role of black and Hispanic physicians in providing health care for underserved populations. The New England journal of medicine. 1996;334(20):1305-10.
23. Marrast LM, Zallman L, Woolhandler S, Bor DH, McCormick D. Minority physicians' role in the care of underserved patients: diversifying the physician workforce may be key in addressing health disparities. JAMA Intern Med. 2014;174(2):289-91.
24. Garcia JA, Paterniti DA, Romano PS, Kravitz RL. Patient preferences for physician characteristics in university-based primary care clinics. Ethnicity & disease. 2003;13(2):259-67.
25. Saha S, Taggart SH, Komaromy M, Bindman AB. Do patients choose physicians of their own race? Health affairs (Project Hope). 2000;19(4):76-83.
26. LaVeist TA, Nickerson KJ, Bowie JV. Attitudes about racism, medical mistrust, and satisfaction with care among African American and white cardiac patients. Medical care research and review : MCRR. 2000;57 Suppl 1:146-61.
27. Laveist TA, Nuru-Jeter A. Is doctor-patient race concordance associated with greater satisfaction with care? Journal of health and social behavior. 2002;43(3):296-306.
28. Cooper-Patrick L, Gallo JJ, Gonzales JJ, Vu HT, Powe NR, Nelson C, et al. Race, gender, and partnership in the patient-physician relationship. Jama. 1999;282(6):583-9.
29. Dunlap JL, Jaramillo JD, Koppolu R, Wright R, Mendoza F, Bruzoni M. The effects of language concordant care on patient satisfaction and clinical understanding for Hispanic pediatric surgery patients. Journal of pediatric surgery. 2015;50(9):1586-9.
30. Ngo-Metzger Q, Sorkin DH, Phillips RS, Greenfield S, Massagli MP, Clarridge B, et al. Providing high-quality care for limited English proficient patients: the importance of language concordance and interpreter use. Journal of general internal medicine. 2007;22 Suppl 2:324-30.
31. Eskes C, Salisbury H, Johannsson M, Chene Y. Patient satisfaction with language--concordant care. The journal of physician assistant education : the official journal of the Physician Assistant Education Association. 2013;24(3):14-22.
32. Hall YN, Xu P, Chertow GM, Himmelfarb J. Characteristics and performance of minority-serving dialysis facilities. Health services research. 2014;49(3):971-91.
33. Almachraki F, Tuffli M, Lee P, Desmarais M, Shih HC, Nissenson AR, et al. Socioeconomic Status of Counties Where Dialysis Clinics Are Located Is an Important Factor in Comparing Dialysis Providers. Population health management. 2016;19(1):70-6.
34. Saunders MR, Lee H, Chin MH. Early winners and losers in dialysis center pay-for-performance. BMC health services research. 2017;17(1):816.
35. Saunders MR, Chin MH. Variation in dialysis quality measures by facility, neighborhood, and region. Medical care. 2013;51(5):413-7.

36. Navaneethan SD, Jolly SE, Schold JD, Arrigain S, Nakhoul G, Konig V, et al. Pragmatic Randomized, Controlled Trial of Patient Navigators and Enhanced Personal Health Records in CKD. Clin J Am Soc Nephrol. 2017;12(9):1418-27.
37. Sullivan C, Leon JB, Sayre SS, Marbury M, Ivers M, Pencak JA, et al. Impact of navigators on completion of steps in the kidney transplant process: a randomized, controlled trial. Clin J Am Soc Nephrol. 2012;7(10):1639-45.
38. Sullivan CM, Barnswell KV, Greenway K, Kamps CM, Wilson D, Albert JM, et al. Impact of Navigators on First Visit to a Transplant Center, Waitlisting, and Kidney Transplantation: A Randomized, Controlled Trial. Clin J Am Soc Nephrol. 2018;13(10):1550-5.
39. Marlow NM, Kazley AS, Chavin KD, Simpson KN, Balliet W, Baliga PK. A patient navigator and education program for increasing potential living donors: a comparative observational study. Clin Transplant. 2016;30(5):619-27.
40. Basu M, Petgrave-Nelson L, Smith KD, Perryman JP, Clark K, Pastan SO, et al. Transplant Center Patient Navigator and Access to Transplantation among High-Risk Population: A Randomized, Controlled Trial. Clin J Am Soc Nephrol. 2018;13(4):620-7.
41. Cervantes L, Chonchol M, Hasnain-Wynia R, Steiner JF, Havranek E, Hull M, et al. Peer Navigator Intervention for Latinos on Hemodialysis: A Single-Arm Clinical Trial. J Palliat Med. 2019.
42. Sullivan C, Dolata J, Barnswell KV, Greenway K, Kamps CM, Marbury Q, et al. Experiences of Kidney Transplant Recipients as Patient Navigators. Transplantation proceedings. 2018;50(10):3346-50.
43. United States Renal Data System. Chapter 1: Incidence, prevalence, patient characteristics, and treatment modalities. Table 1.2 Adjusted prevalence of dialysis, per million, and percentage distribution of diabetes and ethnicity among prevalent dialysis patients, by ESRD network, 2012. Figure 1.6 Trends in (a) prevalent ESRD cases and (b) the adjusted prevalence of ESRD, per million/year, by Hispanic ethnicity, in the US. population, 1996-2012. Bethesda, MD: National Institutes of Health, National Institute of Diabetes and Digestive and Kidney Diseases; 2014 [2014:[Available from: http://www.usrds.org/2014/view/v2_01.aspx.
44. Berlim MT, Mattevi BS, Duarte AP, Thome FS, Barros EJ, Fleck MP. Quality of life and depressive symptoms in patients with major depression and end-stage renal disease: a matched-pair study. J Psychosom Res. 2006;61(5):731-4.
45. Drayer RA, Piraino B, Reynolds CF, 3rd, Houck PR, Mazumdar S, Bernardini J, et al. Characteristics of depression in hemodialysis patients: symptoms, quality of life and mortality risk. Gen Hosp Psychiatry. 2006;28(4):306-12.
46. Hedayati SS, Bosworth HB, Kuchibhatla M, Kimmel PL, Szczech LA. The predictive value of self-report scales compared with physician diagnosis of depression in hemodialysis patients. Kidney Int. 2006;69(9):1662-8.
47. DiMatteo MR, Lepper HS, Croghan TW. Depression is a risk factor for noncompliance with medical treatment: meta-analysis of the effects of anxiety and depression on patient adherence. Arch Intern Med. 2000;160(14):2101-7.
48. Cukor D, Rosenthal DS, Jindal RM, Brown CD, Kimmel PL. Depression is an important contributor to low medication adherence in hemodialyzed patients and transplant recipients. Kidney Int. 2009;75(11):1223-9. doi: 10.038/ki.2009.51. Epub Feb 25.
49. Saran R, Bragg-Gresham JL, Rayner HC, Goodkin DA, Keen ML, Van Dijk PC, et al. Nonadherence in hemodialysis: associations with mortality, hospitalization, and practice patterns in the DOPPS. Kidney Int. 2003;64(1):254-62.
50. Kutner NG, Zhang R, McClellan WM, Cole SA. Psychosocial predictors of non-compliance in haemodialysis and peritoneal dialysis patients. Nephrol Dial Transplant. 2002;17(1):93-9.

51. Lacson E, Jr., Bruce L, Li NC, Mooney A, Maddux FW. Depressive affect and hospitalization risk in incident hemodialysis patients. Clin J Am Soc Nephrol. 2014;9(10):1713-9. doi: 10.2215/CJN.01340214. Epub 2014 Oct 2.
52. Hedayati SS, Grambow SC, Szczech LA, Stechuchak KM, Allen AS, Bosworth HB. Physician-diagnosed depression as a correlate of hospitalizations in patients receiving long-term hemodialysis. Am J Kidney Dis. 2005;46(4):642-9.
53. Boulware LE, Liu Y, Fink NE, Coresh J, Ford DE, Klag MJ, et al. Temporal relation among depression symptoms, cardiovascular disease events, and mortality in end-stage renal disease: contribution of reverse causality. Clin J Am Soc Nephrol. 2006;1(3):496-504. Epub 2006 Mar 1.
54. Fischer MJ, Kimmel PL, Greene T, Gassman JJ, Wang X, Brooks DH, et al. Elevated depressive affect is associated with adverse cardiovascular outcomes among African Americans with chronic kidney disease. Kidney Int. 2011;80(6):670-8. doi: 10.1038/ki.2011.153. Epub Jun 1.
55. Kop WJ, Seliger SL, Fink JC, Katz R, Odden MC, Fried LF, et al. Longitudinal association of depressive symptoms with rapid kidney function decline and adverse clinical renal disease outcomes. Clin J Am Soc Nephrol. 2011;6(4):834-44. doi: 10.2215/CJN.03840510. Epub 2011 Mar 10.
56. Kimmel PL, Peterson RA, Weihs KL, Simmens SJ, Alleyne S, Cruz I, et al. Multiple measurements of depression predict mortality in a longitudinal study of chronic hemodialysis outpatients. Kidney Int. 2000;57(5):2093-8.
57. Chilcot J, Davenport A, Wellsted D, Firth J, Farrington K. An association between depressive symptoms and survival in incident dialysis patients. Nephrol Dial Transplant. 2011;26(5):1628-34. doi: 10.093/ndt/gfq611. Epub 2010 Oct 4.
58. Lacson E, Jr., Li NC, Guerra-Dean S, Lazarus M, Hakim R, Finkelstein FO. Depressive symptoms associate with high mortality risk and dialysis withdrawal in incident hemodialysis patients. Nephrol Dial Transplant. 2012;27(7):2921-8. doi: 10.1093/ndt/gfr778. Epub 2012 Jan 23.
59. Rosenthal Asher D, Ver Halen N, Cukor D. Depression and nonadherence predict mortality in hemodialysis treated end-stage renal disease patients. Hemodial Int. 2012;16(3):387-93. doi: 10.1111/j.542-4758.2012.00688.x. Epub 2012 Apr 3.
60. Lopes AA, Bragg J, Young E, Goodkin D, Mapes D, Combe C, et al. Depression as a predictor of mortality and hospitalization among hemodialysis patients in the United States and Europe. Kidney Int. 2002;62(1):199-207.
61. Riezebos RK, Nauta KJ, Honig A, Dekker FW, Siegert CE. The association of depressive symptoms with survival in a Dutch cohort of patients with end-stage renal disease. Nephrol Dial Transplant. 2010;25(1):231-6. doi: 10.1093/ndt/gfp383. Epub 2009 Aug 4.
62. Hedayati SS, Bosworth HB, Briley LP, Sloane RJ, Pieper CF, Kimmel PL, et al. Death or hospitalization of patients on chronic hemodialysis is associated with a physician-based diagnosis of depression. Kidney Int. 2008;74(7):930-6. doi: 10.1038/ki.2008.311. Epub Jun 25.
63. Young BA, Von Korff M, Heckbert SR, Ludman EJ, Rutter C, Lin EH, et al. Association of major depression and mortality in Stage 5 diabetic chronic kidney disease. Gen Hosp Psychiatry. 2010;32(2):119-24. doi: 10.1016/j.genhosppsych.2009.11.018. Epub 10 Jan 12.
64. Palmer SC, Vecchio M, Craig JC, Tonelli M, Johnson DW, Nicolucci A, et al. Association between depression and death in people with CKD: a meta-analysis of cohort studies. Am J Kidney Dis. 2013;62(3):493-505. doi: 10.1053/j.ajkd.2013.02.369. Epub Apr 25.
65. Cervantes L, Zoucha, J., Jones, J., Fischer, S. Experiences and values of Latinos with end-stage renal disease: A systematic review of qualitative studies. Nephrology Nursing Journal. 2016;43(6):478-93.

66. Lora CM, Gordon EJ, Sharp LK, Fischer MJ, Gerber BS, Lash JP. Progression of CKD in Hispanics: potential roles of health literacy, acculturation, and social support. Am J Kidney Dis. 2011;58(2):282-90. doi: 10.1053/j.ajkd.2011.05.004.
67. Porter AC, Vijil JC, Jr., Unruh M, Lora C, Lash JP. Health-related quality of life in Hispanics with chronic kidney disease. Transl Res. 2010;155(4):157-63. doi: 10.1016/j.trsl.2009.10.005. Epub Nov 14.
68. Lopes AA, Bragg-Gresham JL, Satayathum S, McCullough K, Pifer T, Goodkin DA, et al. Health-related quality of life and associated outcomes among hemodialysis patients of different ethnicities in the United States: the Dialysis Outcomes and Practice Patterns Study (DOPPS). Am J Kidney Dis. 2003;41(3):605-15.
69. Cervantes L, Jones J, Linas S, Fischer S. Qualitative Interviews Exploring Palliative Care Perspectives of Latinos on Dialysis. Clin J Am Soc Nephrol. 2017;12(5):788-98. doi: 10.2215/CJN.10260916. Epub 2017 Apr 12.
70. Cervantes L, Linas S, Keniston A, Fischer S. Latinos With Chronic Kidney Failure Treated by Dialysis: Understanding Their Palliative Care Perspectives. Am J Kidney Dis. 2016;67(2):344-7. doi: 10.1053/j.ajkd.2015.09.026. Epub Nov 20.
71. Cervantes L, Jones J, Linas S, Fischer S. Qualitative Interviews Exploring Palliative Care Perspectives of Latinos on Dialysis. Clinical journal of the American Society of Nephrology : CJASN. 2017;12(5):788-98.
72. Cervantes L, Linas S, Keniston A, Fischer S. Latinos With Chronic Kidney Failure Treated by Dialysis: Understanding Their Palliative Care Perspectives. American journal of kidney diseases : the official journal of the National Kidney Foundation. 2016;67(2):344-7.
73. Cukor D, Ver Halen N, Asher DR, Coplan JD, Weedon J, Wyka KE, et al. Psychosocial intervention improves depression, quality of life, and fluid adherence in hemodialysis. J Am Soc Nephrol. 2014;25(1):196-206. doi: 10.1681/ASN.2012111134. Epub 2013 Oct 10.
74. Kaveh K, Kimmel PL. Compliance in hemodialysis patients: multidimensional measures in search of a gold standard. Am J Kidney Dis. 2001;37(2):244-66.
75. Kimmel PL, Varela MP, Peterson RA, Weihs KL, Simmens SJ, Alleyne S, et al. Interdialytic weight gain and survival in hemodialysis patients: effects of duration of ESRD and diabetes mellitus. Kidney Int. 2000;57(3):1141-51.
