# Supplementary material for: Community Health Worker Support for Hispanic and Latino Individuals Receiving Hemodialysis: The Navigate-Kidney Randomized Clinical Trial
Source: JAMA Intern Med. 2025 Nov 7;186(1):56–66. doi: 10.1001/jamainternmed.2025.5305 (PMC12595546; doi:10.1001/jamainternmed.2025.5305)
Supplement: Supplement 2. — eFigure 1. Navigate-Kidney Intervention for Individuals with Hemodialysis-Dependent Kidney Failure eTable 1. Study Measure Details eReferences eTable 2. Statistical Analysis Details eTable 3. IDWG Outcome for Patients Receiving Hemodialysis Randomly Assigned to Community Health Worker or Standard Arm, Assuming Different Pre-Intervention eFigure 2. Percent of interdialytic weight gain at the largest site (site 3) with 95% confidence limits, assuming same pre-intervention eFigure 3. Systolic blood pressure at the largest site (site 3) with 95% confidence limits, assuming same pre-intervention, along with smooth curves eFigure 4. Phosphorus at the largest site (site 3) with 95% confidence limits, assuming same pre-intervention, along with smooth curves eTable 4. Patient Activation Measure Level at Follow up for Patients Receiving Hemodialysis Randomly Assigned to Navigate-Kidney or Standard Care eTable 5. Characteristics of Study Population by Enrollment Site (n=139) eTable 6. Baseline Measures by Enrollment Site eFigure 5. Percent of interdialytic weight gain at the largest site (site 3) with 95% confidence limits with smooth curves, assuming same pre-intervention [file jamainternmed-e255305-s002.pdf]

Supplemental Online Content

Cervantes L, Juarez-Colunga E, Powe NR, et al. Community health worker support for Hispanic and Latino individuals receiving hemodialysis: the Navigate-Kidney randomized clinical trial. *JAMA Intern Med*. Published online November 7, 2025. doi:10.1001/jamainternmed.2025.5305

eFigure 1. Navigate-Kidney Intervention for Individuals with Hemodialysis-Dependent Kidney Failure
eTable 1. Study Measure Details
eReferences
eTable 2. Statistical Analysis Details
eTable 3. IDWG Outcome for Patients Receiving Hemodialysis Randomly Assigned to Community Health Worker or Standard Arm, Assuming Different Pre-Intervention
eFigure 2. Percent of interdialytic weight gain at the largest site (site 3) with 95% confidence limits, assuming same pre-intervention
eFigure 3. Systolic blood pressure at the largest site (site 3) with 95% confidence limits, assuming same pre-intervention, along with smooth curves
eFigure 4. Phosphorus at the largest site (site 3) with 95% confidence limits, assuming same pre-intervention, along with smooth curves
eTable 4. Patient Activation Measure Level at Follow up for Patients Receiving Hemodialysis Randomly Assigned to Navigate-Kidney or Standard Care
eTable 5. Characteristics of Study Population by Enrollment Site (n=139)
eTable 6. Baseline Measures by Enrollment Site
eFigure 5. Percent of interdialytic weight gain at the largest site (site 3) with 95% confidence limits with smooth curves, assuming same pre-intervention

This supplemental material has been provided by the authors to give readers additional information about their work.

© 2025 Cervantes L et al. *JAMA Intern Med*.

Supplemental Figure 1: Navigate-Kidney Intervention For Individuals with Hemodialysis-Dependent Kidney Failure

Community Health Worker

Characteristics:
- Service-oriented – approachable and accessible
- Empathy and compassion
- Shared lived experiences
- Strong interpersonal skills and communication
- Other: Basic health knowledge, problem-solving skills, team collaboration

Expertise:
- Service-Related field
- High school GED

Training:
- CHW fundamental courses: Motivational interviewing, patient activation, mental health first aid, kidney disease, care coordination, health insurance basics, mental health first aid, and trauma-informed care
- Clinical shadowing: interdisciplinary nephrology clinicians
- Research training

Supervision and Mentorship:
- Weekly CHW team meeting with CHW manager to review challenging cases
- Weekly CHW team meeting with nephrology physician to review patient cases and any clinical or kidney-related questions
- Assess CHW patient load to inform recruitment pause thereby avoiding CHW burnout

Intervention Functions

Build Trust Through Understanding of Health Experience
- Use motivational interviewing and actively listen and empathize
- CHWs will be consistent and reliable by following through on commitments, appointments, and support

Address Multi-Level Social and Structural Challenges to Facilitate Health System Navigation
- CHWs will use social needs screening form and ask patients during first visit which social need is most distressing or impacting their health
- CHWs will collaborate with patients to identify practical solutions for top social needs

Provide Patient-Centered Education
- CHWs will use motivational interviewing techniques and explore the patient's understanding of kidney failure self care including dietary and fluid restriction
- Demonstrate or provide educational materials that are tailored to explain kidney failure, hemodialysis, and dietary restriction

Enhance Self-Management
- CHWs will engage patients using motivational interviewing techniques such as asking open-ended questions, reflective listening, and affirmations, to explore their goals and encourage active participation in managing their kidney care
- CHWs will guide patients in articulating their preferences and concerns, and support them in discussing with clinicians

© 2025 Cervantes L et al. *JAMA Intern Med*.

eTable 1. Study Measure Details

Measure Type	Measure	Description	Study-specific Measurement
Primary outcome	Interdialytic weight gain (IDWG)	Accumulated fluid from salt and fluid intake between dialysis sessions.	Calculated as the difference in patients' weight at the beginning of each HD session (pre-weight) minus the weight after (post-weight) the previous HD session, divided by the prescribed estimated dry weight (EDW), expressed as the percentage of change (IDWG=(pre-weight – post-weight)/dry weight (EDW),%). Measured for 90 days prior to randomization and followed for 180 days after completion of the intervention.
**Hemodialysis adherence outcomes**			
Secondary outcome	Shortened hemodialysis sessions by ≥ 10 minutes[1]	Shortened sessions are tracked by dialysis centers and important for patient safety, quality of care, identifying issues (e.g., identifying patient discomfort), adjusting treatment plan, and regulatory compliance.	Measured for 90 days prior to randomization and followed for 180 days after completion of the intervention.<br><br>Calculated as the rate of shortened hemodialysis sessions by ≥ 10 minutes per 30 days.
Secondary outcome	Pre-dialysis blood pressure[2]	Pre-dialysis systolic blood pressure is collected before the initiation of a dialysis session. Helps in evaluating a patient's volume status, cardiovascular risk, guide antihypertensive therapy, and predict mortality risk.	Measured for 90 days prior to randomization and followed for 180 days after completion of the intervention. Expressed as the systolic blood pressure value (mmHg).
Secondary outcome	Serum phosphorus[3]	Phosphorus is checked every 1 to 3 months. Monitored for interventions such as dietary modification, phosphate binders, and adjustment in dialysis prescription to mitigate risks. High levels are associated with cardiovascular disease, vascular calcification, and bone disorders.	Measured for 90 days prior to randomization and followed for 180 days after completion of the intervention. Expressed as the serum phosphorous value (mg/dL).
**Healthcare utilization outcomes**			
Secondary outcome	Hospitalization	Admission to the inpatient or observational hospital setting.	Binary indicator (yes/no) of occurrence measured for 90 days prior to randomization and followed for 90 days after completion of the intervention.
Secondary outcome	Emergency department visits	Evaluation in an emergency department setting.	Binary indicator (yes/no) of occurrence measured for 90 days prior to randomization and followed for 90 days after completion of the intervention.

© 2025 Cervantes L et al. *JAMA Intern Med.*

**Patient-reported outcomes**			
Secondary Outcome	Patient Activation Measure (PAM)[4]	Assesses patient knowledge, skills and confidence in managing their health	Evaluated at baseline and at closing visit. Expressed as percent of patients at each activation level, 1-4.
Secondary outcome	Renal Adherence Attitudes Questionnaire[5]	A measure of attitudes towards adherence and includes 26 items.	Evaluated at baseline and at closing visit. Calculated as the sum of the 26 questionnaire items.
Secondary outcome	Kidney Dialysis Quality of Life Short Form - 36[6]	A measure of quality of life and assess both general health-related quality of life and specific issues related to kidney disease. Provides a kidney summary score that includes: burden of kidney disease, symptoms and problems, and effects of kidney disease as well a composite for physical health and mental health.	Evaluated at baseline and at closing visit. Kidney summary score is calculated as the average of the 24 items from the burden of kidney disease, symptoms and problems, and effects of kidney disease scales. Physical health and mental health are assessed as composite measures.
**Socio-economic outcomes**			
Secondary outcome	Questions from the Protocol for Responding to and Assessing Patient Assets, Risks, and Experiences (PRAPARE) and from the Accountable Health Communities (AHC) Health Related Social Needs Screening Tool [7,8]	PRAPARE is a standardized tool to assess social challenges in clinical settings and includes 22 core measures that cover domains such as housing stability, employment, education, and more. The AHC focuses on identify health-related social needs and has five core domains including housing instability, food insecurity, transportation difficulties, and more. A mix of questions from PRAPARE and AHC were utilized for this study.	Evaluated at baseline and at closing visit. Expressed as an improvement in social determinants of health (yes/no) at follow up than baseline.

© 2025 Cervantes L et al. *JAMA Intern Med*.

eReferences

1. Cukor D, Ver Halen N, Asher DR, et al. Psychosocial intervention improves depression, quality of life, and fluid adherence in hemodialysis. J Am Soc Nephrol 2014;25(1):196-206. (In eng). DOI: 10.1681/asn.2012111134.
2. Robinson BM, Tong L, Zhang J, et al. Blood pressure levels and mortality risk among hemodialysis patients in the Dialysis Outcomes and Practice Patterns Study. Kidney Int 2012;82(5):570-80. (In eng). DOI: 10.1038/ki.2012.136.
3. KDIGO 2024 Clinical Practice Guideline for the Evaluation and Management of Chronic Kidney Disease. Kidney Int 2024;105(4s):S117-s314. (In eng). DOI: 10.1016/j.kint.2023.10.018.
4. Hibbard JH, Stockard J, Mahoney ER, Tusler M. Development of the Patient Activation Measure (PAM): Conceptualizing and Measuring Activation in Patients and Consumers. Health Services Research 2004;39(4p1):1005-1026. DOI: https://doi.org/10.1111/j.1475-6773.2004.00269.x.
5. Rushe H, McGee HM. Assessing adherence to dietary recommendations for hemodialysis patients: The renal adherence attitudes questionnaire (Raaq) And the renal adherence behaviour questionnaire (Rabq). Journal of Psychosomatic Research 1998;45(2):149-157. DOI: https://doi.org/10.1016/S0022-3999(97)00228-6.
6. Hays RD, Kallich JD, Mapes DL, Coons SJ, Carter WB. Development of the Kidney Disease Quality of Life (KDQOLTM) Instrument. Quality of Life Research 1994;3(5):329-338. DOI: 10.1007/BF00451725.
7. National Association of Community Health Centers, Inc., Association of Asian Pacific Community Health Organizations, and Oregon Primiary Care Association. Protocol for Responding to and Assessing Patient Assets, Risks, and Experiences (PRAPARE). . (https://prapare.org/wp-content/uploads/2023/01/PRAPARE-English.pdf).
8. Centers for Medicare and Medicaid Services. Accountable Health Communities Model. Accountable Health Communities Tool and Guides. . (https://innovation.cms.gov/innovation-models/ahcm).

© 2025 Cervantes L et al. *JAMA Intern Med.*

eTable 2. Statistical Analysis Details

The primary analysis allowed intervention and control group slopes to differ during the 180 days following the completion of the intervention. The primary hypothesis was tested using a likelihood ratio test, comparing the models with and without an interaction between time and intervention group, which allows for different trends/slopes in the post intervention period. For the primary analysis we compared, using Akaike information criteria (AIC), PLMM with and without random slopes for days. The model using random slopes was favored with an AIC of 51,373 vs 51,482 for the model using only random intercepts.<br><br>Logistic regression for secondary outcomes of social risks was performed for each variable to assess whether improvements were associated with receiving the intervention.

© 2025 Cervantes L et al. *JAMA Intern Med*.

eTable 3. IDWG Outcome for Patients Receiving Hemodialysis Randomly Assigned to Community Health Worker or Standard Arm, Assuming Different Pre-Intervention

	Intervention N=68	Control N=71	Difference	P-value
Outcome[1]				
IDWG, mean (CI):				
At 90 days	3.44 (2.93, 3.95)	3.65 (3.16, 4.14)	−0.21 (−0.77, 0.35)	0.004[2]
At 180 days	3.30 (2.83, 3.76)	3.73 (3.28, 4.18)	−0.43 (−0.91, 0.05)	
At 270 days	3.15 (2.69, 3.61)	3.81 (3.36, 4.25)	−0.66 (−1.14, −0.18)	
Difference between 180 and 90 days, mean (CI)	−0.15 (−0.28, −0.01)	0.08 (−0.06, 0.21)	-0.22 (-0.41, -0.03)	
Difference between 270 and 90 days, mean (CI)	−0.29 (−0.56, −0.02)	0.16 (−0.11, 0.42)	-0.45 (-0.83, -0.06)	
Difference between 270 and 180 days, mean (CI)	−0.15 (−0.28, −0.01)	0.08 (−0.06, 0.21)	-0.22 (-0.41, -0.03)	

[1] A piecewise linear mixed model (PLMM) adjusting for enrollment site with random intercepts for patients and random slopes for days has been used. The knot is at the start of the intervention period (at 90 days). The expected mean of the outcomes at the different days for the largest site have been obtained using appropriate contrasts based on the PLMM.
[2] The p-value corresponds to the likelihood ratio test of the interaction between time and intervention arm in the post intervention period.

© 2025 Cervantes L et al. *JAMA Intern Med.*

eFigure 2. Percent of interdialytic weight gain at the largest site (site 3) with 95% confidence limits, assuming same pre-intervention

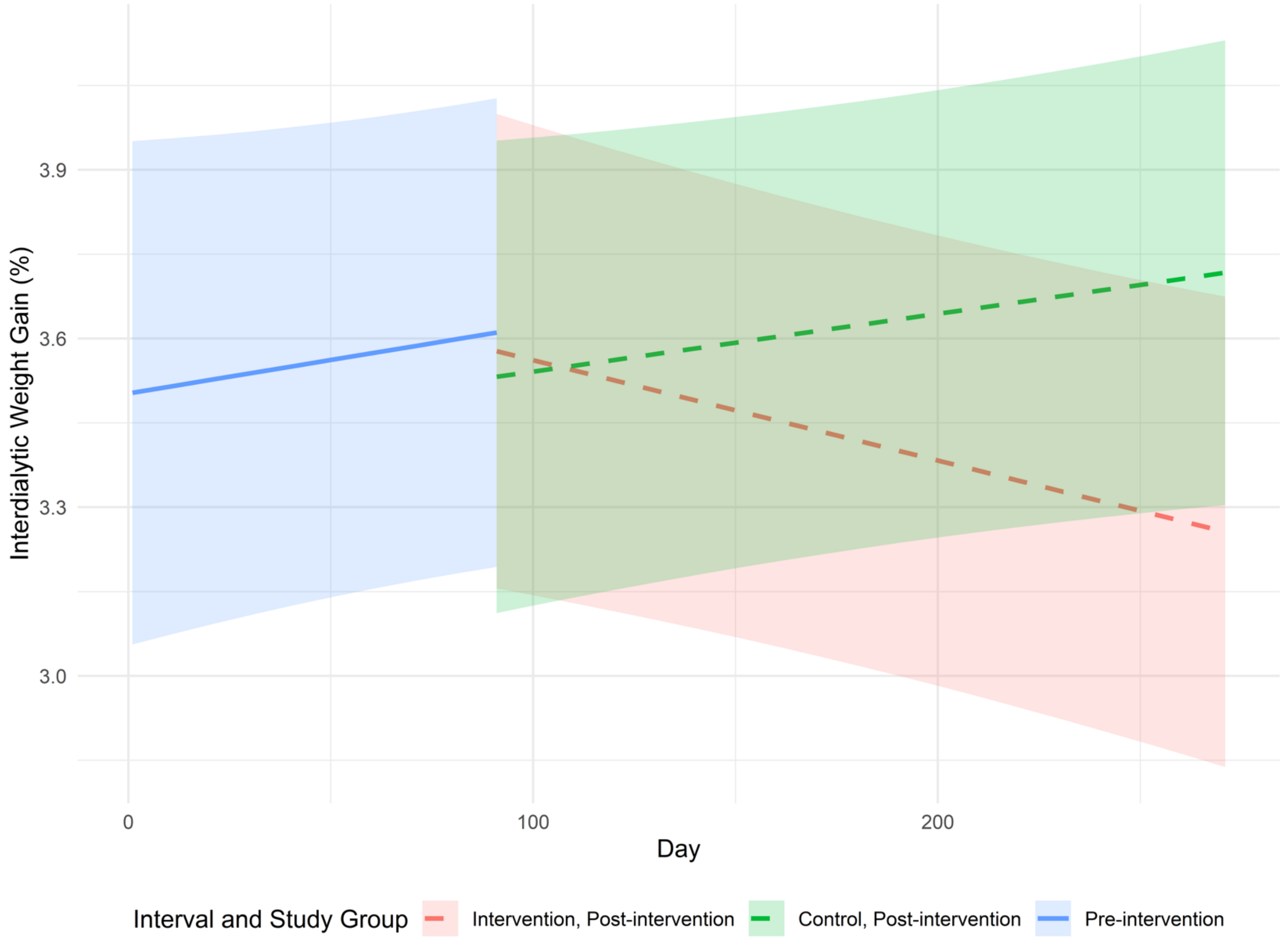

© 2025 Cervantes L et al. *JAMA Intern Med.*

eFigure 3. Systolic blood pressure at the largest site (site 3) with 95% confidence limits, assuming same pre-intervention, along with smooth curves.

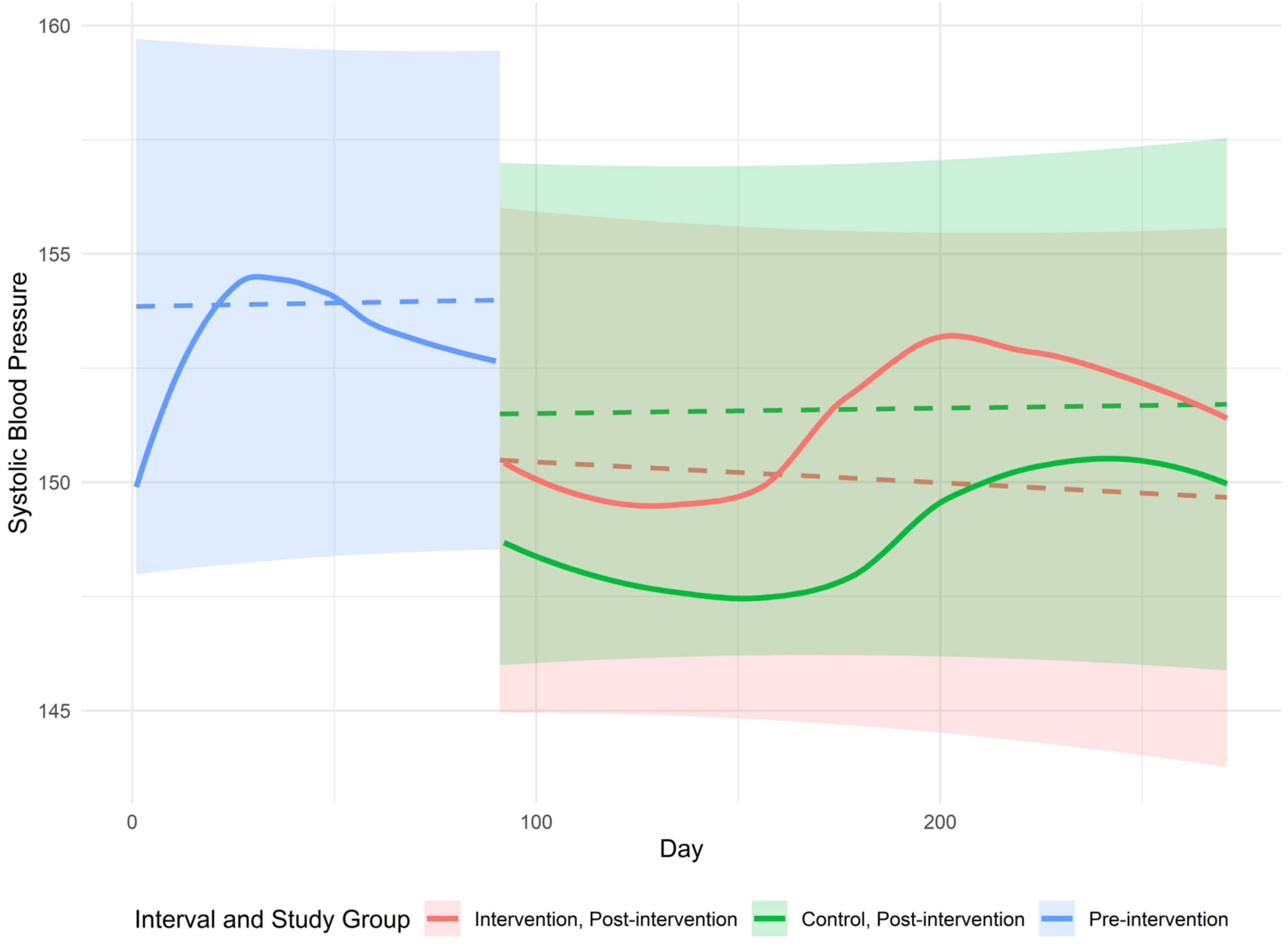

© 2025 Cervantes L et al. *JAMA Intern Med*.

eFigure 4. Phosphorus at the largest site (site 3) with 95% confidence limits, assuming same pre-intervention, along with smooth curves.

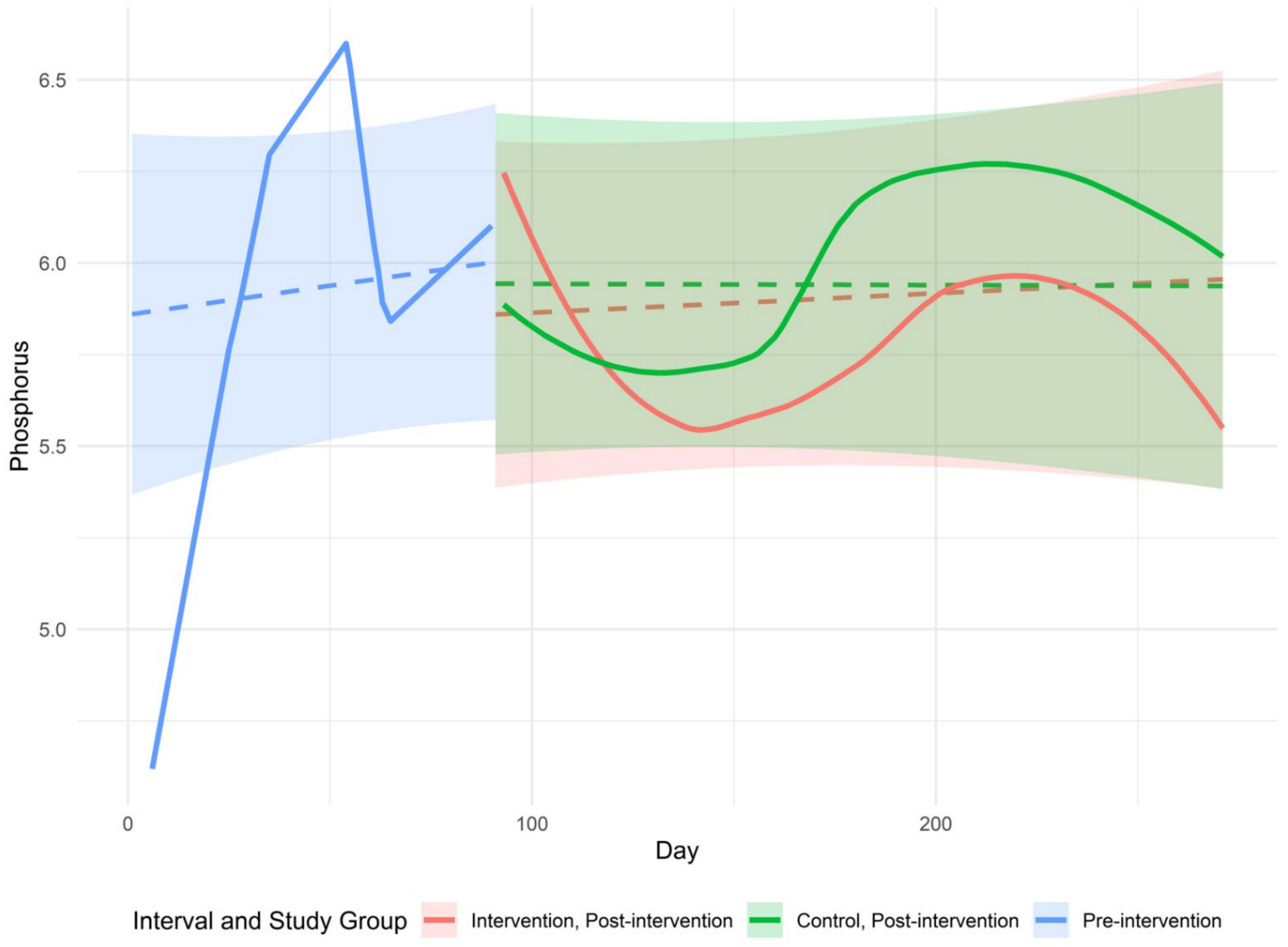

Note: The pre-intervention smooth curve “jumps” early on because measurements are sparse and non-consecutive at site 3 (for example, a single value at day 6 then the next at day 25)—LOESS has to interpolate across large gaps.

© 2025 Cervantes L et al. *JAMA Intern Med*.

eTable 4. Patient Activation Measure Level at Follow up for Patients Receiving Hemodialysis Randomly Assigned to Navigate-Kidney or Standard Care

	Intervention N=68	Control N=71	P-value
Patient Activation Measure (PAM) Level at follow up[3] , No. (%)			Wilcoxon Rank Sum[4]
1	11 (16%)	10 (14%)	0.04
2	19 (28%)	30 (43%)	
3	24 (35%)	20 (29%)	
4	14 (21%)	10 (14%)	

[3] 1 missing
[4] Wilcoxon rank sum test comparing the change in PAM levels from baseline to follow-up between intervention groups has been used.

© 2025 Cervantes L et al. *JAMA Intern Med*.

eTable 5. Characteristics of Study Population by Enrollment Site (n=139)

	No. (%)					
	**Overall (N=139)**	**Site 1 (N=18)**	**Site 2 (N=31)**	**Site 3 (N=50)**	**Site 4 (N=26)**	**Site 5 (N=14)**
**Age in years (SD) mean**	57 (13)	62 (12)	54 (14)	57 (11)	53 (16)	61 (9)
**Female**	68 (49%)	14 (78%)	13 (42%)	20 (40%)	15 (58%)	6 (43%)
**Self-identified Hispanic or Latino**	139 (100%)	18 (100%)	31 (100%)	50 (100%)	26 (100%)	14 (100%)
**Self-identified and Self-reported Race**						
White	21 (15%)	0 (0%)	3 (10%)	8 (16%)	9 (35%)	1 (7%)
American Indian/Alaska Native	8 (6%)	0 (0%)	2 (6%)	4 (8%)	2 (8%)	0 (0%)
Native Hawaiian	1 (1%)	0 (0%)	1 (1%)	0 (0%)	0 (0%)	0 (0%)
Self-identified as Latino/Hispanic or no race (White, Asian, Black, AI/AN, PI/NH)	109 (78%)	18 (100%)	25 (81%)	38 (76%)	15 (58%)	13 (93%)
**Country of origin**						
Mexico	106 (76%)	15 (83%)	23 (74%)	40 (80%)	19 (73%)	9 (64%)
Other (El Salvador, Honduras, Perú)	6 (4%)	0 (0%)	1 (3%)	2 (4%)	3 (12%)	0 (0%)
United States of America	27 (19%)	3 (17%)	7 (23%)	8 (16%)	4 (15%)	5 (36%)
**In general, read and speak language in Spanish**	114 (82%)	14 (78%)	25 (81%)	43 (86%)	22 (85%)	10 (71%)
**How well do you speak English**						
Very Well	26 (19%)	4 (22%)	6 (19%)	8 (16%)	4 (15%)	4 (29%)
Well	18 (13%)	2 (11%)	4 (13%)	5 (10%)	5 (19%)	2 (14%)
Not well	61 (44%)	5 (28%)	13 (42%)	28 (56%)	11 (42%)	4 (29%)
Not at all	34 (24%)	7 (39%)	8 (26%)	9 (18%)	6 (23%)	4 (29%)

© 2025 Cervantes L et al. *JAMA Intern Med.*

	No. (%)					
	**Overall (N=139)**	**Site 1 (N=18)**	**Site 2 (N=31)**	**Site 3 (N=50)**	**Site 4 (N=26)**	**Site 5 (N=14)**
**Highest level of school finished**						
More than high school	20 (14%)	4 (22%)	3 (10%)	6 (12%)	3 (12%)	4 (29%)
High school diploma or GED	16 (12%)	0 (0%)	5 (16%)	6 (12%)	3 (12%)	2 (14%)
Less than high school	103 (74%)	14 (78%)	23 (74%)	38 (76%)	20 (77%)	8 (57%)
**Current work situation[5]**						
Full time work	5 (4%)	0 (0%)	1 (3%)	3 (6%)	1 (4%)	0 (0%)
Part-time or temporary work	20 (15%)	2 (11%)	2 (6%)	11 (22%)	5 (22%)	0 (0%)
Unemployed	111 (82%)	16 (89%)	28 (90%)	36 (72%)	17 (74%)	14 (100%)
**Past year total combined income for participant and the family members they live with[6]**						
< $25,000	96 (72%)	8 (47%)	26 (84%)	41 (82%)	18 (69%)	3 (30%)
Over $25,000	22 (16%)	1 (6%)	3 (10%)	9 (18%)	7 (27%)	2 (20%)
Don't know/Choose not to answer	16 (12%)	8 (47%)	2 (6%)	0 (0%)	1 (4%)	5 (50%)
**Insurance**						
Dual Medicare/Medicaid	47 (34%)	5 (28%)	12 (39%)	19 (38%)	6 (23%)	5 (36%)
Medicaid	34 (24%)	3 (17%)	8 (26%)	13 (26%)	7 (27%)	3 (21%)
Medicare	6 (4%)	2 (11%)	0 (0%)	3 (6%)	0 (0%)	1 (7%)
Other public or Private	52 (37%)	8 (44%)	11 (35%)	15 (30%)	13 (50%)	5 (36%)

[5] 3 missing
[6] 5 missing

© 2025 Cervantes L et al. *JAMA Intern Med*.

	No. (%)					
	**Overall (N=139)**	**Site 1 (N=18)**	**Site 2 (N=31)**	**Site 3 (N=50)**	**Site 4 (N=26)**	**Site 5 (N=14)**
**In the past year, have you or any family members you live with been unable to get:**						
Food	35 (25%)	8 (44%)	10 (32%)	10 (20%)	4 (15%)	3 (21%)
Clothing	32 (23%)	6 (33%)	9 (29%)	10 (20%)	6 (23%)	1 (7%)
Utilities	48 (35%)	7 (39%)	13 (42%)	15 (30%)	7 (27%)	6 (43%)
Childcare	7 (5%)	1 (6%)	2 (6%)	2 (4%)	1 (4%)	1 (7%)
Medicine or any healthcare	42 (30%)	7 (39%)	9 (29%)	14 (28%)	8 (31%)	4 (29%)
Cell Phone	29 (21%)	3 (17%)	9 (29%)	9 (18%)	5 (19%)	3 (21%)
**In the past 12 months, electric, gas, oil, or water company threatened to shut off services**[7]	25 (18%)	4 (22%)	8 (28%)	7 (14%)	2 (8%)	4 (29%)
**In the past 12 months, there were days that the home was not heated/cooled because bills were not paid**	11 (8%)	1 (6%)	5 (16%)	1 (2%)	1 (4%)	3 (21%)
**In the past 12 months, how many times did you decide not to fill or refill a prescription because it was too expensive**						
1 time	15 (11%)	4 (22%)	5 (16%)	3 (6%)	2 (8%)	1 (7%)
2 times	13 (9%)	4 (22%)	2 (6%)	6 (12%)	0 (0%)	1 (7%)
3-4 times	17 (12%)	2 (11%)	4 (13%)	7 (14%)	2 (8%)	2 (14%)
None	94 (68%)	8 (44%)	20 (65%)	34 (68%)	22 (85%)	10 (71%)

[7] 2 missing

© 2025 Cervantes L et al. *JAMA Intern Med.*

	No. (%)					
	**Overall (N=139)**	**Site 1 (N=18)**	**Site 2 (N=31)**	**Site 3 (N=50)**	**Site 4 (N=26)**	**Site 5 (N=14)**
**Mode of transport to and from dialysis[8]**						
Benefit transportation (Medicaid)	38 (28%)	6 (33%)	6 (19%)	13 (27%)	6 (23%)	7 (54%)
Family or friend	35 (26%)	8 (44%)	7 (23%)	11 (22%)	7 (27%)	2 (15%)
I drive	48 (35%)	3 (17%)	13 (42%)	21 (43%)	9 (35%)	2 (15%)
Public transportation	16 (12%)	1 (6%)	5 (16%)	4 (8%)	4 (15%)	2 (15%)
**Within the past 12 months, we worried whether our food would run out before we got money to buy more**						
Never true	81 (58%)	9 (50%)	19 (61%)	27 (54%)	15 (58%)	11 (79%)
Often true	11 (8%)	3 (17%)	3 (10%)	4 (8%)	1 (4%)	0 (0%)
Sometimes true	47 (34%)	6 (33%)	9 (29%)	19 (38%)	10 (38%)	3 (21%)
**Within the past 12 months, the food we bought just didn't last and we didn't have money to get more**						
Never true	87 (63%)	11 (61%)	21 (68%)	29 (59%)	16 (62%)	10 (71%)
Often true	12 (9%)	3 (17%)	3 (10%)	5 (10%)	1 (4%)	0 (0%)
Sometimes true	40 (29%)	4 (22%)	7 (23%)	16 (32%)	9 (35%)	4 (29%)
**Within the past 12 months, we couldn't afford to eat balanced meals**						
Never true	71 (51%)	10 (56%)	18 (58%)	21 (42%)	12 (46%)	10 (71%)

[8] 2 missing

© 2025 Cervantes L et al. *JAMA Intern Med.*

	No. (%)					
	**Overall (N=139)**	**Site 1 (N=18)**	**Site 2 (N=31)**	**Site 3 (N=50)**	**Site 4 (N=26)**	**Site 5 (N=14)**
Often true	12 (9%)	2 (11%)	3 (10%)	4 (8%)	2 (8%)	1 (7%)
Sometimes true	56 (40%)	6 (33%)	10 (32%)	25 (50%)	12 (46%)	3 (21%)
**Are you worried about losing your housing?**	39 (28%)	7 (39%)	12 (39%)	14 (28%)	4 (15%)	2 (14%)
**Patient Activation Measure (PAM) Level**						
1 – Disengaged and overwhelmed	25 (18%)	5 (28%)	5 (16%)	10 (20%)	4 (15%)	1 (7%)
2 – Becoming aware but struggling	49 (35%)	9 (50%)	19 (61%)	10 (20%)	5 (19%)	6 (43%)
3 – Taking action	33 (24%)	1 (6%)	6 (19%)	15 (30%)	6 (23%)	5 (36%)
4 – Maintaining behaviors and pushing further	32 (23%)	3 (17%)	1 (3%)	15 (30%)	11 (42%)	2 (14%)
**Months on dialysis, median (IQR)**	34.0 (12.0, 64.0)	45.0 (24.3, 69.5)	24.0 (12.0, 54.0)	38.0 (15.5, 72.0)	29.5 (11.0, 60.0)	11.5 (4.5, 34.8)
**Distance between home and dialysis center in miles, median (IQR)[9]**	5.0 (3.0, 8.0)	8.0 (6.0, 8.0)	5.0 (3.0, 6.5)	4.0 (3.0, 7.0)	4.0 (3.0, 8.8)	4.0 (2.0, 7.0)
**Travel time between home and dialysis center in minutes, median (IQR)[10]**	15.0 (10.0, 25.0)	20.0 (17.8, 25.0)	12.0 (10.0, 20.0)	15.0 (10.0, 20.0)	15.0 (12.8, 28.8)	15.0 (15.0, 20.0)
**KDQOL Kidney summary score, mean (SD)[11]**	65.4 (17.0)	66.9 (15.2)	65.7 (17.9)	66.7 (16.9)	61.8 (15.9)	64.4 (20.5)

[9] 2 missing
[10] 2 missing
[11] Higher KDQOL scores means better quality of life.

© 2025 Cervantes L et al. *JAMA Intern Med.*

	No. (%)					
	**Overall (N=139)**	**Site 1 (N=18)**	**Site 2 (N=31)**	**Site 3 (N=50)**	**Site 4 (N=26)**	**Site 5 (N=14)**
**KDQOL Burden of kidney disease, mean (SD)**	35.9 (23.1)	38.2 (21.7)	40.7 (26.9)	32.6 (20.1)	31.3 (18.0)	42.4 (31.9)
**KDQOL Symptoms/problems, mean (SD)**	74.0 (16.8)	76.3 (13.5)	73.2 (15.4)	77.6 (16.6)	68.4 (19.3)	70.5 (17.4)
**KDQOL Effects of kidney disease, mean (SD)**	67.1 (23.4)	67.2 (22.7)	67.0 (23.4)	67.4 (25.0)	67.1 (17.2)	66.3 (30.8)
**KDQOL SF-12 Physical health composite, mean (SD)**	35.0 (9.8)	34.5 (7.9)	34.7 (8.9)	35.1 (10.9)	35.1 (9.6)	35.5 (10.7)
**KDQOL SF-12 Mental health composite, mean (SD)**	50.6 (10.2)	49.5 (11.6)	51.6 (10.1)	50.7 (10.3)	49.9 (9.9)	50.9 (10.4)
**Brief health literacy score, mean (SD)**	9.7 (5.0)	12.2 (5.3)	10.4 (3.7)	7.7 (4.5)	9.4 (5.6)	12.4 (5.2)
**Including yourself, how many family members, do you currently live with? median (IQR)**	3.0 (2.0, 5.0)	3.5 (3.0, 4.0)	4.0 (2.5, 5.0)	2.5 (1.0, 4.8)	4.0 (2.0, 5.0)	3.0 (2.3, 4.0)
**Serum Albumin, mean (SD)[12]**	4 (0.5)	3.9 (0.2)	4.0 (0.3)	4.2 (0.7)	3.9 (0.4)	4.0 (0.4)
**Serum Potassium, mean (SD)[13]**	4.8 (0.8)	4.9 (0.8)	4.8 (0.8)	4.8 (0.8)	4.9 (0.9)	4.6 (0.5)
**Serum Phosphorus, mean (SD)[14]**	5.5 (1.9)	4.8 (1.6)	5.7 (2.3)	6.1 (1.9)	4.9 (1.7)	5.2 (0.9)
**CCI Score, mean (SD)[15]**	2.8 (1.7)	NA (NA)	2.2 (1.9)	3.3 (1.5)	2.1 (1.8)	3.0 (NA)

[12] 24 missing
[13] 6 missing
[14] 5 missing
[15] 51 missing

© 2025 Cervantes L et al. *JAMA Intern Med.*

	No. (%)					
	**Overall (N=139)**	**Site 1 (N=18)**	**Site 2 (N=31)**	**Site 3 (N=50)**	**Site 4 (N=26)**	**Site 5 (N=14)**
**Follow-Up Period after the intervention in days, median (IQR)**	182.0 (179.0, 182.5)	182.0 (182.0, 182.0)	180.0 (176.0, 182.0)	182.0 (180.3, 183.8)	180.0 (179.0, 182.8)	179.0 (43.0, 181.5)

Abbreviations: GED, General Educational Development, KDQOL Kidney Dialysis Quality of Life, SF Short Form, CCI Charlson Comorbidity Index

© 2025 Cervantes L et al. *JAMA Intern Med*.

eTable 6. Baseline Measures by Enrollment Site

	Site 1 (N=18)	Site 2 (N=31)	Site 3 (N=50)	Site 4 (N=26)	Site 5 (N=14)
**Primary Outcome**					
Baseline IDWG, mean (SD)	3.55 (3.09)	2.98 (1.52)	4.25 (2.21)	4.10 (2.98)	3.71 (1.77)
**Secondary outcome: Hemodialysis adherence measures**					
Pre Rate of Missed Dialysis Sessions (per 30 days), median (IQR)	0.00 (0.00, 0.33)	0.33 (0.00, 0.66)	0.00 (0.00, 0.33)	0.00 (0.00, 0.33)	0.00 (0.00, 0.00)
Pre Rate of Shortened Dialysis Sessions (per 30 days), median (IQR)	0.34 (0.33, 1.64)	1.65 (0.66, 3.83)	1.97 (0.99, 2.97)	1.48 (0.68, 2.03)	0.99 (0.42, 2.23)
Baseline Pre-dialysis Sitting Systolic Blood Pressure, mean (SD)	160.78 (26.86)	154.23 (24.91)	152.64 (22.59)	150.00 (25.34)	155.21 (24.97)
Baseline Phosphorus[16], mean (SD)	4.81 (1.57)	5.73 (2.28)	6.08 (1.87)	4.93 (1.67)	5.22 (0.93)
**Secondary outcomes: patient-centered assessments**					
Baseline Renal Adherence Attitudes **Questionnaire**, median (IQR)	16.50 (11.25, 21.00)	18.00 (13.00, 23.00)	18.00 (11.25, 28.50)	18.00 (15.00, 21.75)	16.00 (11.25, 24.75)
Baseline Patient Activation Measure, median (IQR)	49.95 (47.48, 53.20)	51.00 (51.00, 53.20)	60.60 (51.00, 72.50)	65.40 (51.00, 75.00)	54.40 (51.00, 62.48)

Abbreviations: IDWG, Interdialytic weight gain

[16] 5 missing

© 2025 Cervantes L et al. *JAMA Intern Med.*

eFigure 5. Percent of interdialytic weight gain at the largest site (site 3) with 95% confidence limits with smooth curves, assuming same pre-intervention

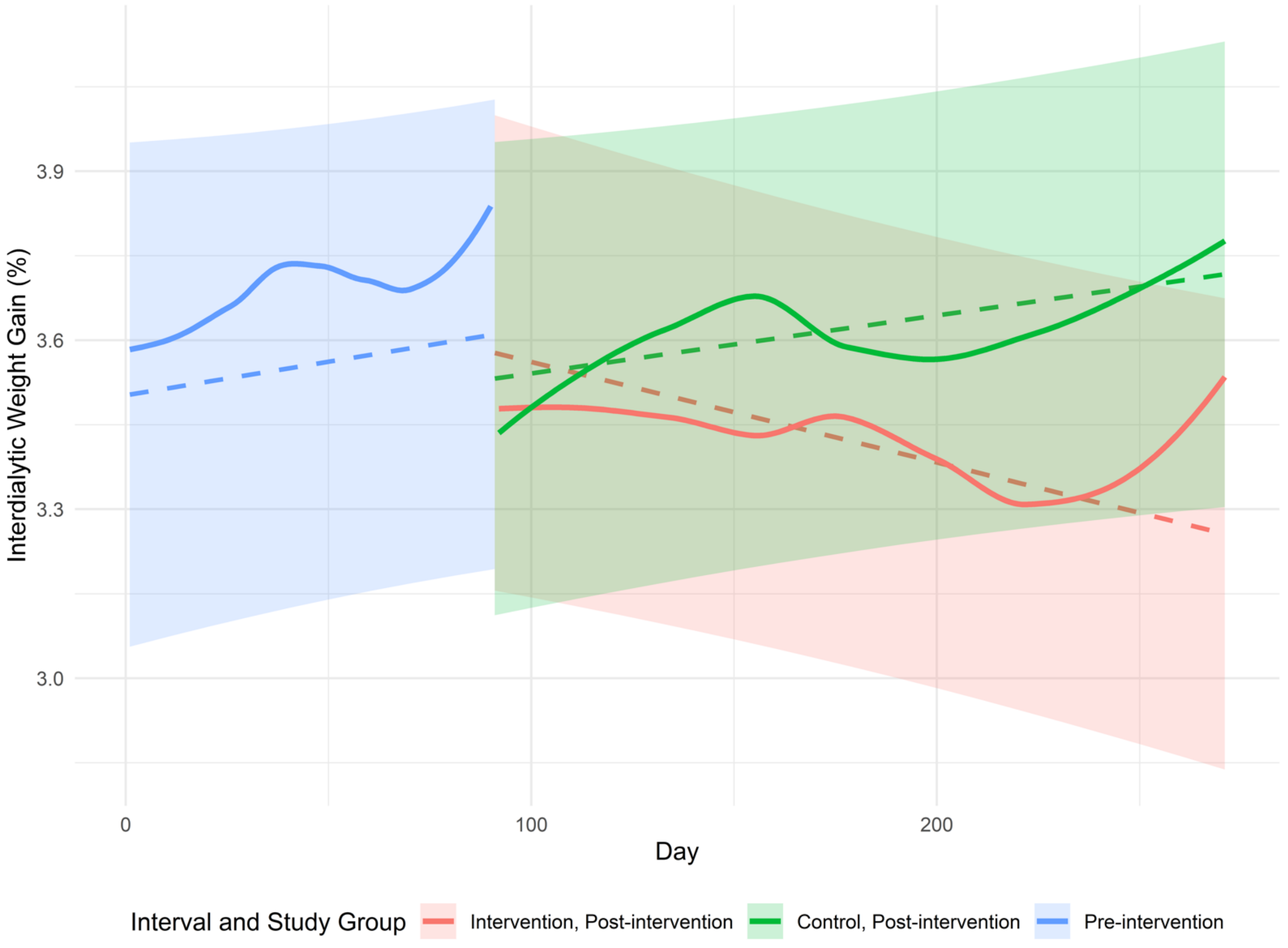

© 2025 Cervantes L et al. *JAMA Intern Med*.
