# Supplementary material for: Community Health Worker Support for Hispanic and Latino Individuals Receiving Hemodialysis: The Navigate-Kidney Randomized Clinical Trial
Source: JAMA Intern Med. 2025 Nov 7;186(1):56–66. doi: 10.1001/jamainternmed.2025.5305 (PMC12595546; doi:10.1001/jamainternmed.2025.5305)
Supplement: Supplement 3. — Data Sharing Statement [file jamainternmed-e255305-s003.pdf]

# Data Sharing Statement

Cervantes. Community Health Worker Support for Hispanic and Latino Individuals Receiving Hemodialysis. *JAMA Intern Med*. Published November 07, 2025.
doi:10.1001/jamainternmed.2025.5305

**Data**
**Additional Information:** Trial Registration: ClinicalTrials.gov Identifier: NCT03978806
**Data available:** Yes
**Data types:** Deidentified participant data
**How to access data:** The data will be shared through the NIDDK central repository 12 months after publication of the main paper.
**When available:** beginning date: 08-01-2027

**Supporting Documents**
**Document types:** None

**Additional Information**
**Who can access the data:** It will be managed and available through the NIDDK central repository.
**Types of analyses:** For any purpose.
**Mechanisms of data availability:** They will be managed and available through the NIDDK central repository.
